# Synthesis of aliphatic nitriles from cyclobutanone oxime mediated by sulfuryl fluoride (SO_2_F_2_)

**DOI:** 10.3762/bjoc.19.68

**Published:** 2023-06-22

**Authors:** Xian-Lin Chen, Hua-Li Qin

**Affiliations:** 1 School of Chemistry, Chemical Engineering and Life Science, Wuhan University of Technology, 205 Luoshi Road, Wuhan, 430070, PR China

**Keywords:** direct N–O activation, *E*-selectivity, nitrile synthesis, ring-opening cross-coupling, sulfuryl fluoride, SO_2_F_2_

## Abstract

A SO_2_F_2_-mediated ring-opening cross-coupling of cyclobutanone oxime derivatives with alkenes was developed for the construction of a range of δ-olefin-containing aliphatic nitriles with (*E*)-configuration selectivity. This new method features wide substrate scope, mild conditions, and direct N–O activation.

## Introduction

**Scheme 1:**
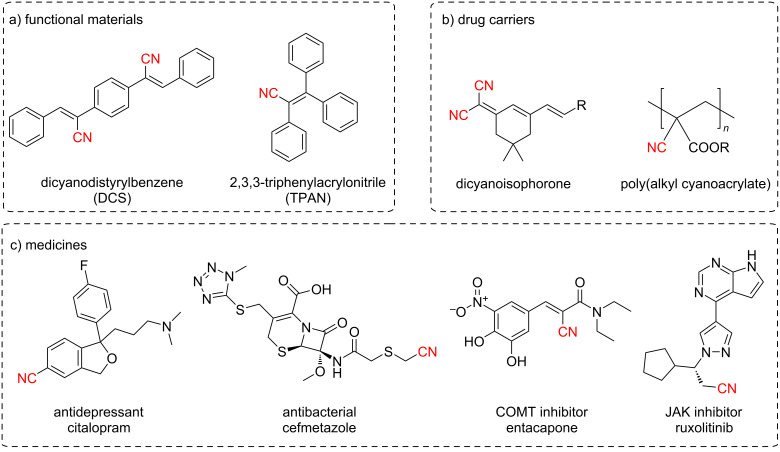
Representative nitrile-containing functional materials, drug carriers, and medicines.

As an important functional group in organic molecules, the nitrile group is commonly present in functional materials [1–2], nanoscale drug carriers [3–5], biologically valuable molecules and drugs (Scheme 1) [6–7]. There are over 70 nitrile-containing drugs approved by the FDA for various indications and more than 140 additional nitrile-containing leads in clinical investigation [8]. Looking into changing the physicochemical properties, in the field of drug discovery, it is important to explore solutions to introduce nitrile groups into a molecule for enhancing the interaction between the drug candidate and the target protein, to further improve the efficacy of the potential drug [9]. The nitrile group can also function as a metabolic blocking site to inhibit the oxidative metabolism of molecules to improve metabolic stability in vivo [10]. Consequently, the development of novel synthetic methods and strategies toward nitrile group construction continues to be a focus for synthetic chemists.

The cross-coupling reactions of C–C bonds catalyzed by transition-metal complexes play a crucial role in modern organic synthesis, as they make it feasible to synthesize complex structures from available components [11–13]. Indeed, the formation of C(sp^2^)–C(sp^3^) bonds by cross-coupling has developed rapidly in recent years [14–16], but it still remains less advanced than the synthesis of C(sp^2^)–C(sp^2^) bonds [17–18]. This is attributed to the electron-richness of the C(sp^3^) carbon, which leads to side reactions of the alkyl intermediates [14,19–20]. Besides, most of the C(sp^2^)–C(sp^3^) reactions employ organic halides or organometallic reagents [21–23], which are not environmentally friendly.

**Scheme 2:**
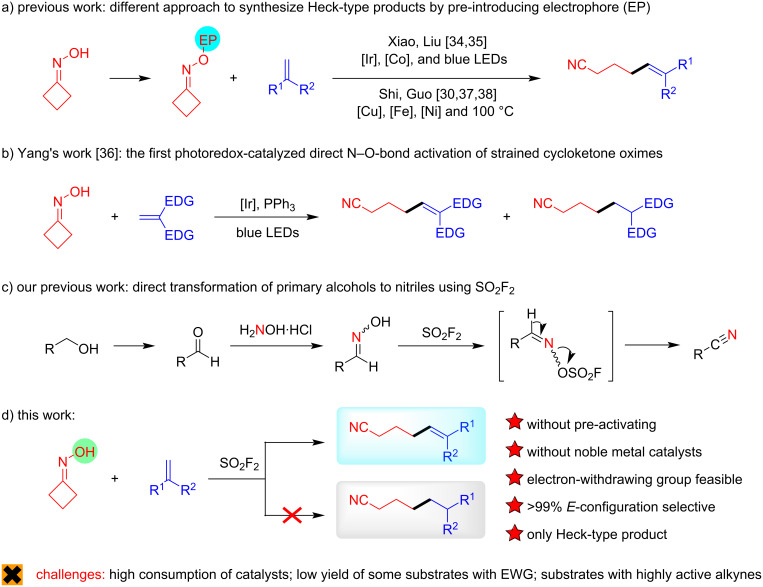
Activating protocol of cyclobutanone oximes.

Recently, based on the activation effect of *O*-acyloximes on N–O bonds [24–29], a synthesis method for δ-olefin-containing aliphatic nitriles by the radical C–C bond cleavage of cycloketone oxime ester derivatives was developed by Shi’s group (Scheme 2a) [30], which emerged as an efficient strategy to construct C(sp^2^)–C(sp^3^) bonds [31–33]. Later, Xiao [34], Liu [35], and Yang [36] achieved similar transformations through visible-light photocatalysis. In addition, Guo [37–38] improved the protocol by using low-cost nickel and iron catalysts. However, most of these advancements mainly relied on the excellent redox potential manipulation of cyclic oxime esters and adopted the pre-acylation activation strategies [39–41]. Up to now, only one report employed an oxime for the generation of iminyl radicals to obtain the similar products, in which, substrates were limited to the electron-rich alkenes (Scheme 2b) [42].

On the other hand, sulfuryl fluoride (SO_2_F_2_) [43], a kind of inexpensive (about 1 $/kg), abundant, and relatively inert electrophile and one of the major sulfur fluoride exchange (SuFEx) click chemistry reagents [44–45], has been successfully applied as an electrophile to react with hydroxy groups to generate fluorosulfonate esters, being activated intermediates for a variety of transformations [46–58]. Lately, we discovered the SO_2_F_2_-mediated transformation of primary alcohols to nitriles, involving an aldoxime sulfonyl ester intermediate (Scheme 2c) [59]. Drawing inspiration from these excellent works, we contemplated that the N–O bond of cyclobutanone oxime derivatives could be activated by SO_2_F_2_ in situ to enable cleavage of the C–C bond, which could achieve this transformation without going through inefficient pre-introduction of electrophores. Herein, we describe how this concept has been translated into experimental reality, developing a new SO_2_F_2_-mediated C–C single bond cleavage method for constructing δ-olefin-containing aliphatic nitriles.

## Results and Discussion

We started our investigation by selecting cyclobutanone oxime (**1a**) and 1,1-diphenylethylene (**2a**) as model starting materials to testify the feasibility of this proposed transformation in the presence of *N*,*N*-diisopropylethylamine (DIPEA) and Cu(OTf)_2_ in dioxane/PhCF_3_ (1:1) under an SO_2_F_2_ atmosphere at 100 °C. The desired product 6,6-diphenylhex-5-enenitrile (**3aa**) was obtained in 24% yield (Table 1, entry 1) and according to the control experiment, SO_2_F_2_ is essential for the reaction to proceed (Table 1, entry 2). Encouraged by the preliminary result, we then screened a large variety of conditions as shown in Table 1 in order to improve the efficiency of the transformation. The investigation of the solvent effect revealed that in 1,4-dioxane the transformations performed the best (Table 1, entries 3–5). A series of copper catalysts such as CuI, CuCN, and Cu_2_O was screened, in which some showed good catalytic activity (Table 1, entries 6–9), and Cu_2_O was identified as the most effective catalyst for the desired transformation. Accordingly, the catalyst loading of Cu_2_O was studied next and increasing the loading of Cu_2_O to 1.0 equivalent, gave the desired product **3aa** in a good yield of 72% (Table 1, entry 10). Furthermore, the examination of the effect of base revealed CH_3_COOK being the most suitable choice (Table 1, entry 11). Either increasing the temperature to 120 °C or decreasing to 80 °C resulted in an obviously decreased yield (Table 1, entries 13 and 14), which could probably be attributed to the decomposition of the highly active sulfonyl ester intermediate. The reaction time was also screened and the yield did not change within the accuracy errors when the time was extended (Table 1, entry 15) and among the screened reaction times, 12 hours were chosen as the optimal conditions (see Supporting Information File 1 for more details).

**Table 1:** Screening the optimized reaction conditions.^a^

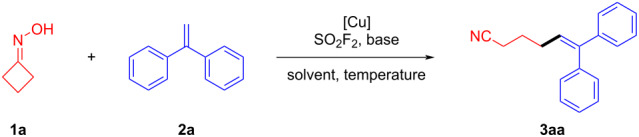					

Entry	[Cu] cat.	Base	Solvent	*T* (°C)	Yield (%)^b^

1^c^	Cu(OTf)_2_ (10 mol %)	DIPEA	dioxane/PhCF_3_	100	24
2^c,d^	Cu(OTf)_2_ (10 mol %)	DIPEA	dioxane/PhCF_3_	100	N.D.
3^c^	Cu(OTf)_2_ (10 mol %)	DIPEA	PhCF_3_	100	N.D.
4^c^	Cu(OTf)_2_ (10 mol %)	DIPEA	1,4-dioxane	100	47
5^c^	Cu(OTf)_2_ (10 mol %)	DIPEA	CH_2_Cl_2_	100	39
6^c^	CuI (10 mol %)	DIPEA	1,4-dioxane	100	41
7^c^	CuCN (10 mol %)	DIPEA	1,4-dioxane	100	40
8^c^	Cu_2_O (10 mol %)	DIPEA	1,4-dioxane	100	55
9^c^	/	DIPEA	1,4-dioxane	100	N.D.
10^c^	Cu_2_O (100 mol %)	DIPEA	1,4-dioxane	100	72
11^c^	Cu_2_O (100 mol %)	CH_3_COOK	1,4-dioxane	100	75
12	Cu_2_O (100 mol %)	CH_3_COOK	1,4-dioxane	100	83
13	Cu_2_O (100 mol %)	CH_3_COOK	1,4-dioxane	80	54
14	Cu_2_O (100 mol %)	CH_3_COOK	1,4-dioxane	120	64
15^e^	Cu_2_O (100 mol %)	CH_3_COOK	1,4-dioxane	100	79

^a^Reaction conditions: A mixture of cyclobutanone oxime (**1a**, 1.5 mmol, 3.0 equiv), 1,1-diphenylethylene (**2a**, 0.5 mmol, 1.0 equiv), copper catalyst and base (5.0 mmol, 10.0 equiv) in extra dry solvent (0.1 M) was stirred at the corresponding temperature under an SO_2_F_2_ atmosphere (balloon) for 12 h. ^b^The yield was determined by HPLC using pure **3aa** as the external standard (*t*_R_ = 5.017 min, λ_max_ = 250.0 nm, water/methanol 20:80 (v/v)). ^c^6.0 equiv of base were used. ^d^Under Ar atmosphere (balloon) instead of SO_2_F_2_. ^e^The reaction lasted 16 h.

**Scheme 3:**
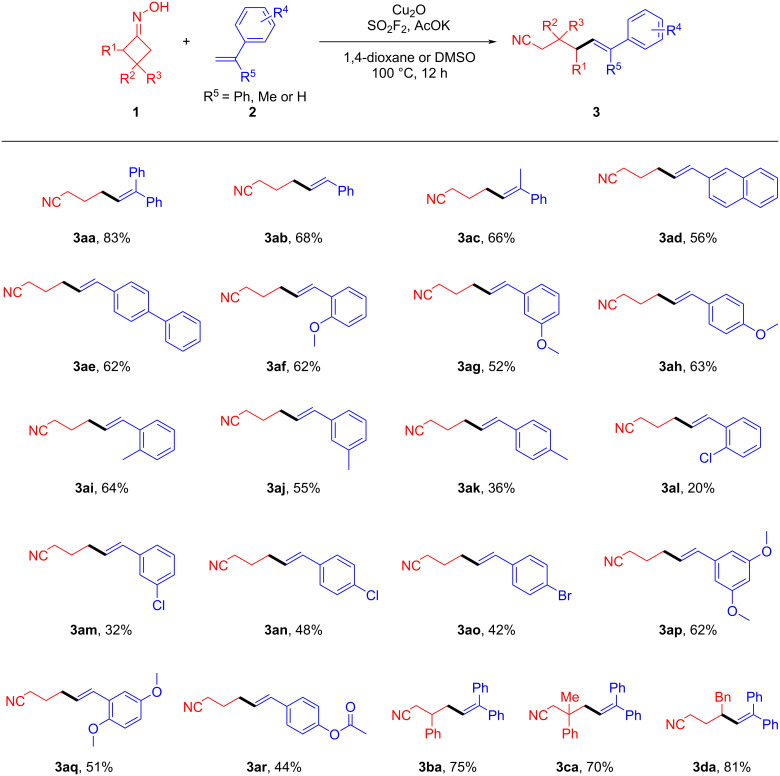
Substrate scope of δ-olefin-containing aliphatic nitriles. Reaction conditions: A mixture of cyclobutanone oxime derivative **1** (3.0 mmol, 3.0 equiv), alkene **2** (1.0 mmol, 1.0 equiv), Cu_2_O (1.0 mmol, 1.0 equiv) and potassium acetate (10.0 mmol, 10.0 equiv) in extra dry dioxane or DMSO (0.1 M) was stirred at 100 °C under an SO_2_F_2_ atmosphere (balloon) for 12 h; yields refer to isolated compounds.

With the optimized reaction conditions in hand, a range of other substrates possessing representative functional groups was employed subsequently to evaluate the reaction scope and limitations (Scheme 3). Under the optimized conditions, alkenes **2b**–**e** with varying steric effects underwent smooth reaction, yielding the corresponding products **3ab**–**ae** in moderate to good yields (56–68%). Notably, the efficiency of this transformation was greatly impacted by the electronic effect on the aromatic rings of the olefins. Alkenes with electron-donating groups on their aromatic rings showed higher yields of the corresponding products as compared to those with electron-withdrawing groups (**3af**–**ar**). Furthermore, the desired products were not even obtained when the starting materials were connected to extra strong electron-withdrawing groups (such as a nitro group) on their aromatic rings. In addition, a series of cyclobutanone oxime derivatives were also smoothly transformed into the corresponding nitriles **3ba**–**da** in excellent yields.

**Scheme 4:**
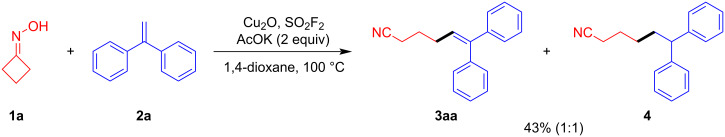
Competition between two reactions caused by the reduction of base equivalent.

Interestingly, when the loading of CH_3_COOK was reduced to 2 equivalents, we obtained a mixture of unsaturated nitrile **3aa** and saturated nitrile **4** after column chromatography (Scheme 4). We speculated that the reduction of the base equivalent may induce the ionization of **1a** and facilitate the ultimate addition process. The selectivity of bases for different processes may attract significant attention for further applications.

**Scheme 5:**
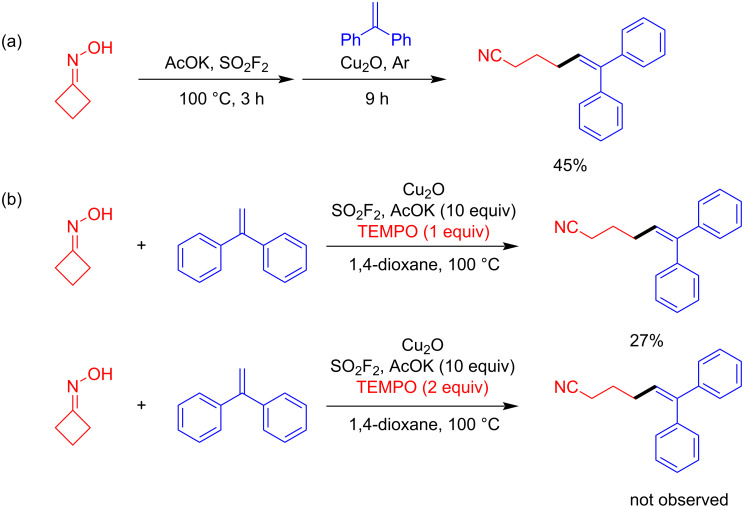
Mechanistic investigations.

In order to understand the mechanism of the aforementioned transformation, some experimental investigations were performed as described in Scheme 5. Under the promotion of the base, cyclobutanone oxime preliminarily reacts with SO_2_F_2_, generating the activated precursor fluorosulfonate, which further reacts with the alkene **2a** in the presence of the copper catalyst under Ar atmosphere for 9 h (Scheme 5a). The corresponding product was successfully obtained in 45% yield, which indirectly proved the existence of an oxime sulfonyl ester intermediate (fluorosulfonate). As shown in Scheme 5b, in the presence of one equivalent of TEMPO, a commonly used radical scavenger, the yield of **3aa** significantly decreased, in addition, the reaction was completely inhibited when the amount of added TEMPO was increased to 2 equivalents.

**Scheme 6:**
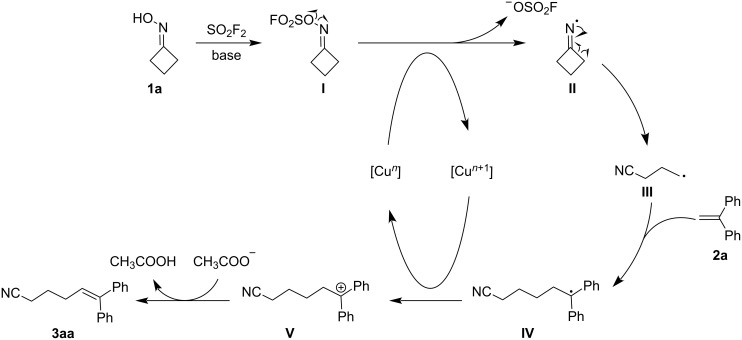
A proposed plausible mechanism.

Based upon the preliminary results and previous reports of this class of transformation [26,30,36–37,42,60–61], a plausible mechanism for the base-promoted, SO_2_F_2_-mediated ring-opening cross-coupling of cyclobutanone oxime derivatives with alkenes was proposed (Scheme 6). Initially, cyclobutanone oxime reacts with SO_2_F_2_, generating an oxime sulfonyl ester intermediate (fluorosulfonate) **I** promoted by the base. Subsequently, the intermediate fluorosulfonate **I** undergoes single-electron reduction by [Cu*^n^*] in situ to afford the iminyl radical intermediate **II**. In the following step, the ring-strain of cyclobutanone is released under the promotion of the imine radical, giving the *C*-centered radical **III** which is subsequently captured by the alkene. Meanwhile, the radical **IV** transfers an electron to [Cu*^n^*^+1^] regenerating the [Cu*^n^*] catalyst and intermediate **V**. The critical β-H elimination step occurs smoothly in the presence of excessive base to generate the final nitrile product. Due to the high reactivity of the intermediate fluorosulfonate I, the attempt of isolation or detecting the in situ generated intermediate I was not accomplished either by NMR analysis or chromatography.

## Conclusion

In conclusion, we have developed an SO_2_F_2_-mediated ring-opening cross-coupling reaction of cyclobutanone oxime derivatives with alkenes for the synthesis of a class of novel elongated nitriles. The newly constructed δ-olefin-containing aliphatic nitriles possess *E*-configuration at the double bond. This transformation could be easily activated by SO_2_F_2_ in situ without the need of pre-introduction of electrophores.

## Supporting Information

File 1 Experimental information.
