# Supplementary material for: Synthesis of aliphatic nitriles from cyclobutanone oxime mediated by sulfuryl fluoride (SO2F2)
Source: Beilstein J Org Chem. 2023 Jun 22;19:901–8. doi: 10.3762/bjoc.19.68 (PMC10291241; doi:10.3762/bjoc.19.68)
Supplement: File 1 — Experimental information. [file Beilstein_J_Org_Chem-19-901-s001.pdf]

BEILSTEIN JOURNAL OF ORGANIC CHEMISTRY

Supporting Information

for

Synthesis of aliphatic nitriles from cyclobutanone oxime mediated by sulfuryl fluoride (SO_2F_2)

Xian-Lin Chen and Hua-Li Qin

Beilstein J. Org. Chem. **2023,** *19,* 901–908. doi:10.3762/bjoc.19.68

Experimental information

License and Terms: This is a supporting information file under the terms of the Creative Commons Attribution License (https://creativecommons.org/licenses/by/4.0). Please note that the reuse, redistribution and reproduction in particular requires that the author(s) and source are credited and that individual graphics may be subject to special legal provisions.
The license is subject to the *Beilstein Journal of Organic Chemistry* terms and conditions: (https://www.beilstein-journals.org/bjoc/terms)

Table of contents

1. General information S2

2. Optimization of the reaction conditions S3

3. General procedures S11

4. Product characterization S20

5. References S27

6. NMR spectra S28

1. General information

All reactions were carried out in dried glassware. All reagents were purchased from commercial sources and used without further purification. Unless otherwise specified, NMR spectra were recorded in $CDCl_3$ on a 500 MHz (for ^{1}H), 471 MHz (for ^{19}F), 126 MHz (for ^{13}C) Bruker Avance spectrometer, and were internally referenced to solvent residual signals (note: $CDCl_3$: δH = 7.264 ppm, δC = 77.16 ppm). The HPLC experiments were carried out on a Waters e2695 instrument (column: J&K, RP-C18, 5 μm, 4.6 × 150 mm), and the yields of the products were determined by using the corresponding pure compounds as the external standards. The coupling constants were reported in hertz (Hz). The following abbreviations were used to explain the multiplicities: s = singlet, d = doublet, t = triplet, q = quartet, m = multiplet, br = broad.

2. Optimization of the reaction conditions

Table S1 Screening the solvent[a]

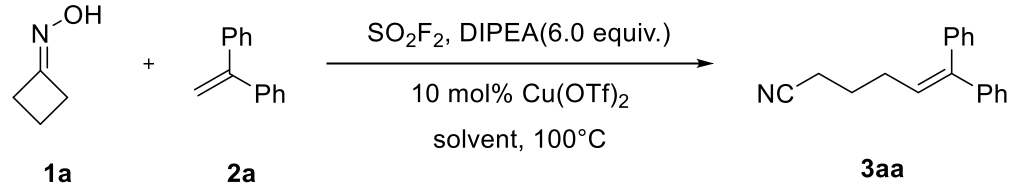

Entry	Solvent	Yield (**3aa**,%)[b]
**1**	**1,4-dioxane**	**47**
2	$PhCF_3$	N.D.
3	DMF	13
4	NMP	23
5	$PhCH_3$	12
6	CH_3CN	N.D.
7	CH_2Cl_2	39
8	THF	N.D.
9	CH_2ClCH_2Cl	N.D.
10	acetone	N.D.
11	dioxane/DMSO (1:1)	21
12	dioxane/$PhCF_3$ (1:1)	24
13	dioxane/NMP (1:1)	32
14	dioxane/CH_2Cl_2 (1:1)	46

[a] Reaction conditions: A mixture of cyclobutanone (**1a**, 1.5 mmol, 3.0 equiv), 1,1-diphenylethylene (**2a**, 0.5 mmol), $Cu(OTf)_2$ (0.05 mmol, 10 mol %) and DIPEA (6.0 equiv) in anhydrous solvent (0.1 M) was stirred at 100 °C with a SO_2F_2 balloon for 12 h. [b] The yield was determined by HPLC using pure **3aa** as the external standard (t_R = 5.0 min, λ_{max} = 250.0 nm, water/methanol 20:80 (v/v)). N.D. = Not detectable.

Table S2 Screening the copper catalyst[a]

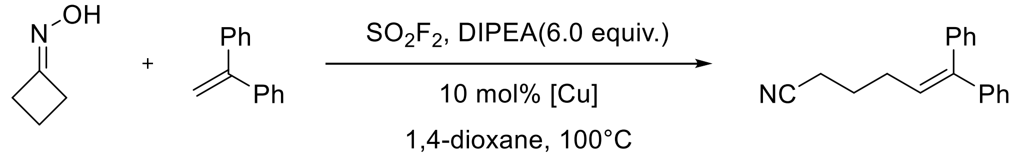

Entry	[Cu]	Yield (**3aa**,%)[b]
1	$Cu(OTf)_2$	47
2	CuO	N.D.
3	CuF_2	15
4	CuI	41
5	$Cu(CH_3CN)_4PF_6$	38
6	Cu power	16
7	Zu Cu alloy	16
8	CuCl	34
9	CuBr	9
**10**	**Cu_2O**	**55**
11	CuCN	40
12	/	N.D.
13	$CuSO_4$	35

[a] Reaction conditions: A mixture of cyclobutanone (**1a**, 1.5 mmol, 3.0 equiv), 1,1-diphenylethylene (**2a**, 0.5 mmol), copper catalyst (0.05 mmol, 10 mol %) and DIPEA (3.0 mmol, 6.0 equiv) in anhydrous 1,4-dioxane (0.1 M) was stirred at 100 °C with a SO_2F_2 balloon for 12 h. [b] The yield was determined by HPLC using pure **3aa** as the external standard (t_R = 5.0 min, λ_{max} = 250.0 nm, water/methanol 20:80 (v/v)). N.D. = Not detectable.

Table S3 Screening the loading of copper catalyst[a]

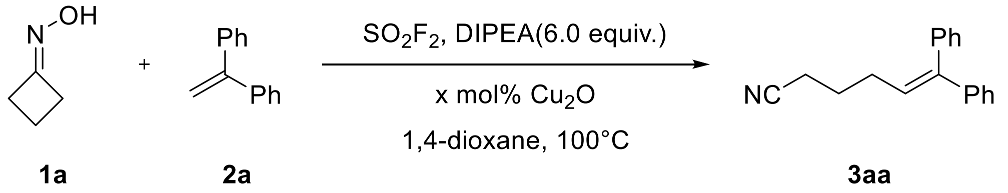

Entry	Cu_2O (x mol %)	Yield (**3aa**,%)[b]
1	10	55
2	30	48
3	50	61
**4**	**100**	**72**
5	120	71
6	150	66
7	200	46

[a] Reaction conditions: A mixture of cyclobutanone (**1a**, 1.5 mmol, 3.0 equiv), 1,1-diphenylethylene (**2a**, 0.5 mmol), Cu_2O (x mol %) and DIPEA (3.0 mmol, 6.0 equiv) in anhydrous 1,4-dioxane (0.1 M) was stirred at 100 °C with a SO_2F_2 balloon for 12 h. [b] The yield was determined by HPLC using pure **3aa** as the external standard (t_R = 5.0 min, λ_{max} = 250.0 nm, water/methanol 20:80 (v/v)).

Table S4 Screening the base[a]

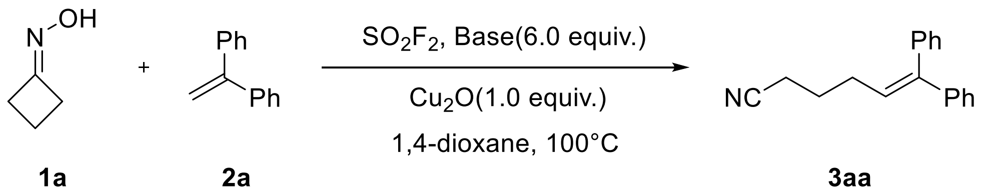

Entry	Base	Yield (**3aa**,%)[b]
1	K_2CO_3	40
2	Et_3N	37
3	DBU	N.D.
4	DIPEA	72
5	Na_2CO_3	62
6	Cs_2CO_3	29
7	$NaHCO_3$	N.D.
8	TMEDA	51
9	Li_2CO_3	N.D.
10	CH_3OK	11
11	KF	N.D.
**12**	**CH_3COOK**	**75**
13	*t*-BuONa	N.D.
14	PMDETA	7

[a] Reaction conditions: A mixture of cyclobutanone (**1a**, 1.5 mmol, 3.0 equiv), 1,1-diphenylethylene (**2a**, 0.5 mmol), Cu_2O (0.5 mmol, 1.0 equiv) and Base (3.0 mmol, 6.0 equiv) in anhydrous 1,4-dioxane (0.1 M) was stirred at 100 °C with a SO_2F_2 balloon for 12 h. [b] The yield was determined by HPLC using pure **3aa** as the external standard (t_R = 5.0 min, λ_{max} = 250.0 nm, water/methanol 20:80 (v/v)). N.D. = Not detectable.

Table S5 Screening the loading of base[a]

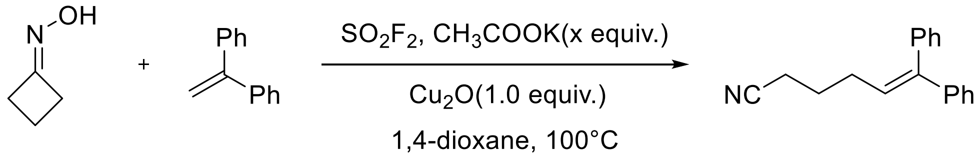

1a **2a** **3aa**

Entry	CH_3COOK (x equiv)	Yield (**3aa**,%)[b]
1	2.0	21
2	3.0	24
3	4.0	23
4	5.0	55
5	6.0	75
6	7.0	65
7	8.0	72
8	9.0	79
**9**	**10.0**	**83**
10	11.0	83
11	12.0	84

[a] Reaction conditions: A mixture of cyclobutanone (**1a**, 1.5 mmol, 3.0 equiv), 1,1-diphenylethylene (**2a**, 0.5 mmol), Cu_2O (0.5 mmol, 1.0 equiv) and CH_3COOK (x equiv) in anhydrous 1,4-dioxane (0.1 M) was stirred at 100 °C with a SO_2F_2 balloon for 12 h. [b] The yield was determined by HPLC using pure **3aa** as the external standard (t_R = 5.0 min, λ_{max} = 250.0 nm, water/methanol 20:80 (v/v)). N.D. = Not detectable.

Table S6 Screening the loading of 1a[a]

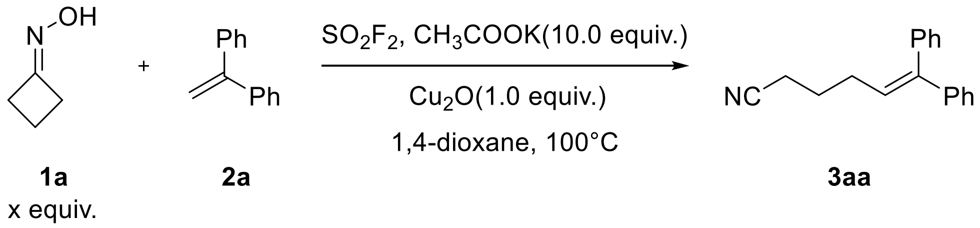

Entry	**1a** (x equiv)	Yield (**3aa**,%)[b]
1	0.33	N.D.
2	0.5	N.D.
3	1.0	15
4	1.5	35
5	2.0	50
**6**	**3.0**	**83**
7	5.0	76

[a] Reaction conditions: A mixture of cyclobutanone (**1a**, x equiv), 1,1-diphenylethylene (**2a**, 0.5 mmol), Cu_2O (0.5 mmol, 1.0 equiv) and CH_3COOK (5.0 mmol, 10.0 equiv) in anhydrous 1,4-dioxane (0.1 M) was stirred at 100 °C with a SO_2F_2 balloon for 12 h. [b]The yield was determined by HPLC using pure **3aa** as the external standard (t_R = 5.0 min, λ_{max} = 250.0 nm, water/methanol 20:80 (v/v)). N.D. = Not detectable.

Table S7 Screening the reaction temperature[a]

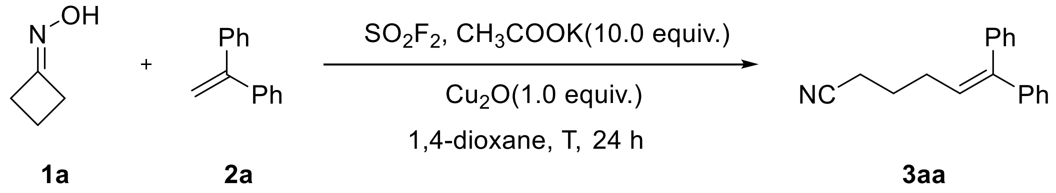

Entry	Temperature (°C)	Yield (**3aa**,%)[b]
1	80	54
**2**	**100**	**83**
3	120	64

[a] Reaction conditions: A mixture of cyclobutanone (**1a**, 1.0 mmol, 3.0 equiv), 1,1-diphenylethylene (**2a**, 0.5 mmol), Cu_2O (0.5 mmol, 1.0 equiv) and CH_3COOK (5.0 mmol, 10.0 equiv) in anhydrous 1,4-dioxane (0.1 M) was stirred at T with a SO_2F_2 balloon for 12 h. [b] The yield was determined by HPLC using pure **3aa** as the external standard (t_R = 5.0 min, λ_{max} = 250.0 nm, water/methanol 20:80 (v/v)).

Table S8 Screening the reaction time[a]

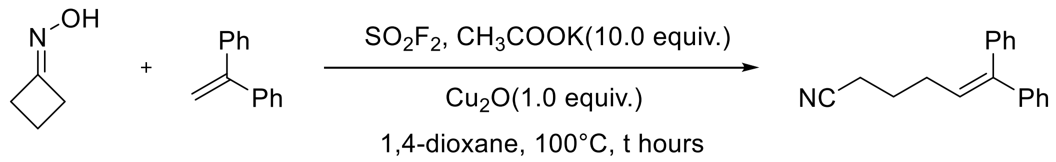

1a **2a** **3aa**

Entry	Time (h)	Yield (**3aa**,%)[b]
1	8	60
**2**	**12**	**83**
3	16	79
4	24	81

[a] Reaction conditions: A mixture of cyclobutanone (**1a**, 1.0 mmol, 3.0 equiv), 1,1-diphenylethylene (**2a**, 0.5 mmol), Cu_2O (100 mol %) and CH_3COOK (10.0 equiv) in anhydrous 1,4-dioxane (0.1 M) was stirred at 100 °C with a SO_2F_2 balloon for t hours. [b] The yield was determined by HPLC using pure **3aa** as the external standard (t_R = 5.0 min, λ_{max} = 250.0 nm, water/methanol 20:80 (v/v)).

3. General procedures

3.1 General procedures for synthesis of the compounds 1[1,2]

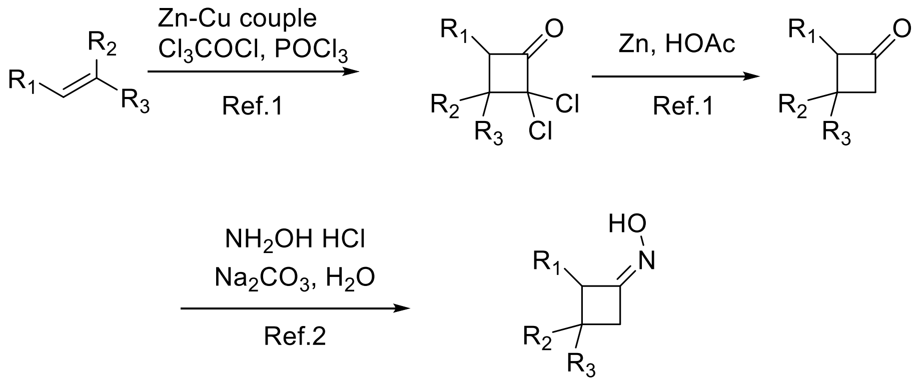

Cyclobutanone derivatives, were either commercially available or produced by the reduction of 2,2-dichlorocyclobutanones synthesized from the corresponding alkenes by the reported procedure[2]. The following experimental procedure is typical: To a 50 mL three-necked flask under argon were added alkene derivative (5.0 mmol, 1.0 equiv), zinc–copper couple (960 mg, 15.0 mmol, 3.0 equiv), and anhydrous ether (10 mL). To this was added a solution of trichloroacetyl chloride (1.12 mL, 10.0 mmol, 2.0 equiv) and phosphorus oxychloride (0.51 mL, 5.5 mmol, 1.1 equiv) in ether (10 mL) over 1 h through an addition funnel. The suspension was stirred overnight at reflux. The resulting mixture was filtered through a pad of Celite and washed with ether (20 mL). The organic solution was successively washed with water (30 mL), a saturated aqueous solution of $NaHCO_3$ (30 mL) and brine (30 mL), and dried over $MgSO_4$. Then, the solution was filtered, concentrated and used in the next step without further purification.

A mixture of 2,2-dichlorocyclobutanones (1.0 equiv) and zinc dust (4.0 equiv) in acetic acid (10 mL) was stirred at room temperature for 2 h and then heated at 80 °C for 5 h. The resulting mixture was allowed to cool to room temperature, then, the solution was diluted with water (30 mL) and extracted with ether (3 × 20 mL). The organic phase was washed successively with a saturated solution of aqueous $NaHCO_3$ (3 × 30 mL), water (30 mL) and brine (30 mL), then dried over $MgSO_4$ and concentrated in vacuum. The crude material was then purified by flash chromatography eluting with a mixture of petroleum ether and ethyl acetate to afford various

cyclobutanones.

To a stirred solution of the cyclobutanone (1.0 equiv) in H_2O (0.5 M) was added hydroxylamine hydrochloride (2.0 equiv) and Na_2CO_3 (0.5 equiv) at 45 ℃. After stirring for 3 h, the residue was diluted with water and extracted with EtOAc. The aqueous layer was extracted with EtOAc and the combined organic extracts were washed with brine, dried over $MgSO_4$, and evaporated under reduced pressure to give the crude material, which was used in the next step without further purification.

3.2 General procedures for synthesis of the compounds 3

Method A:

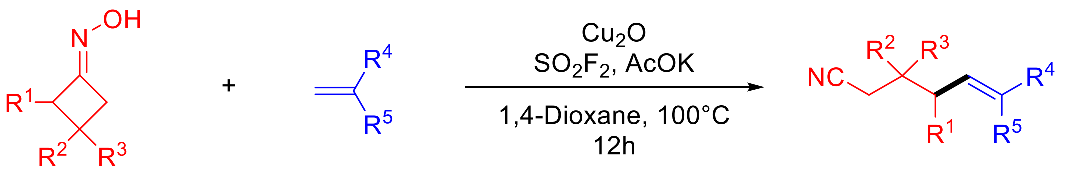

Cu_2O (1.0 mmol), AcOK (10.0 mmol), the cyclobutanone oxime derivative (3.0 mmol), the alkene (1.0 mmol) and extra dry 1,4-dioxane (10 mL) were added to a 50 mL oven-dried round-bottomed flask that was equipped with a stirring bar. The flask was fitted with a plastic stopper and SO_2F_2 gas was introduced into the stirring reaction mixture by bubbling from a SO_2F_2 balloon. The mixture was stirred strongly at 100 °C for 12 h, then cooled to room temperature upon completion. After that, the reaction mixture was filtered with diatomite to remove insoluble substances such as copper catalyst and excessive base, washed by ethyl acetate. Then, the reaction mixture was diluted with water and extracted with ethyl acetate (3 × 15 mL) and the combined organic layers were dried over anhydrous sodium sulfate, filtered and concentrated under reduced pressure. The residue was purified by column chromatography on silica gel to afford the desired nitriles.

Attention: SO_2F_2 has certain inhalation toxicity, please operate in fume hood.
The SO_2F_2 bottle should be stored in a cool, dry, and well ventilated place.

Method B:

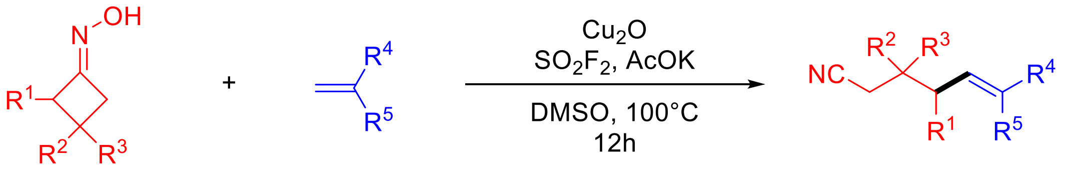

1 2 3

Some substances with low yields have higher yields using Method B. Cu_2O (1.0 mmol), AcOK (10.0 mmol), the cyclobutanone oxime derivative (3.0 mmol), the alkene (1.0 mmol) and extra dry DMSO (10 mL) were added to a 50 mL oven-dried round-bottomed flask that was equipped with a stirring bar. The flask was fitted with a plastic stopper and SO_2F_2 gas was introduced into the stirring reaction mixture by bubbling from a SO_2F_2 balloon. The mixture was stirred strongly at 100 °C for 12 h, then cooled to room temperature upon completion. After that, the reaction mixture was diluted with water and filtered with diatomite, washed by ethyl acetate. Then, the reaction mixture was extracted with ethyl acetate (3 × 15 mL) and the combined organic layers were dried over anhydrous sodium sulfate, filtered and concentrated under reduced pressure. The residue was purified by column chromatography on silica gel to afford the desired nitriles.

Pictures of the procedures for synthesis of the compounds 3:

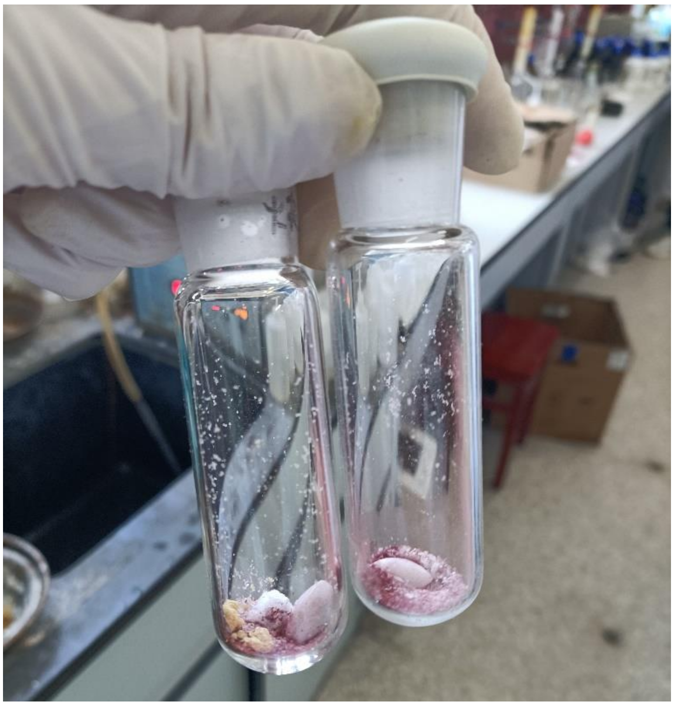

Figure S1: First, add all solid materials into an oven-dried tube or flask

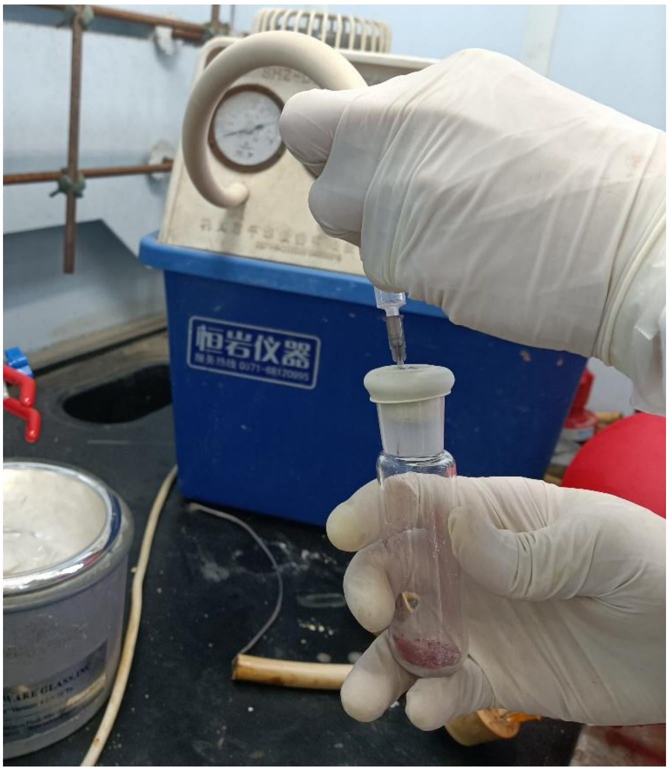

Figure S2: Using a vacuum pump to remove air

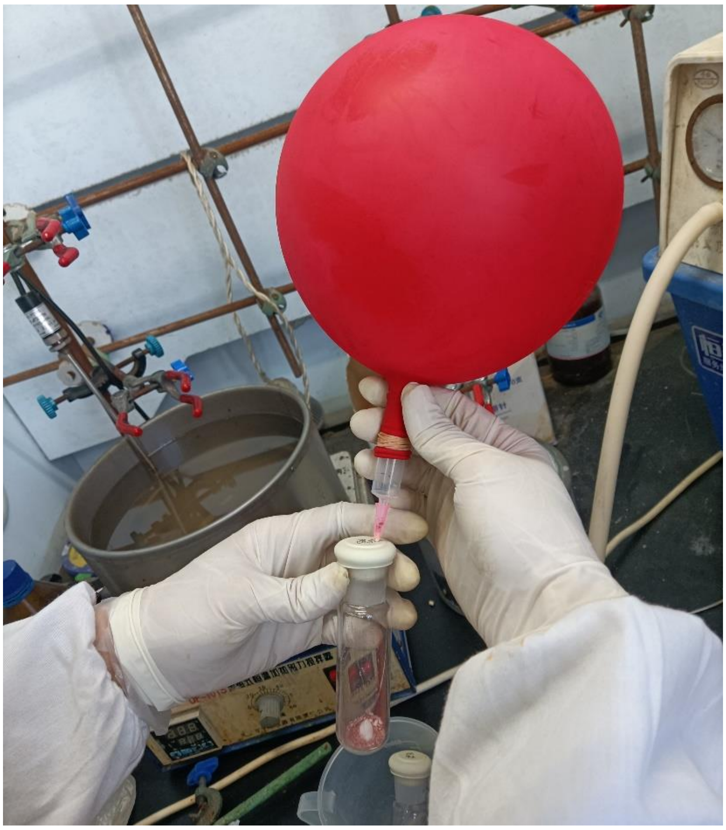

Figure S3: Bubbling from a SO_2F_2 balloon, or extracting and exchanging air for three times.

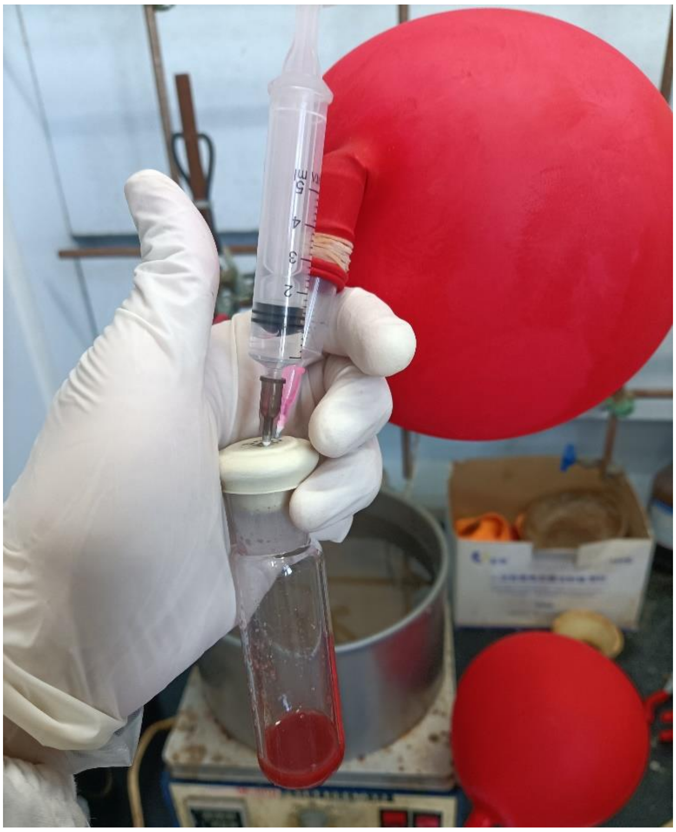

Figure S4: Add all liquid materials into the reaction vessel.

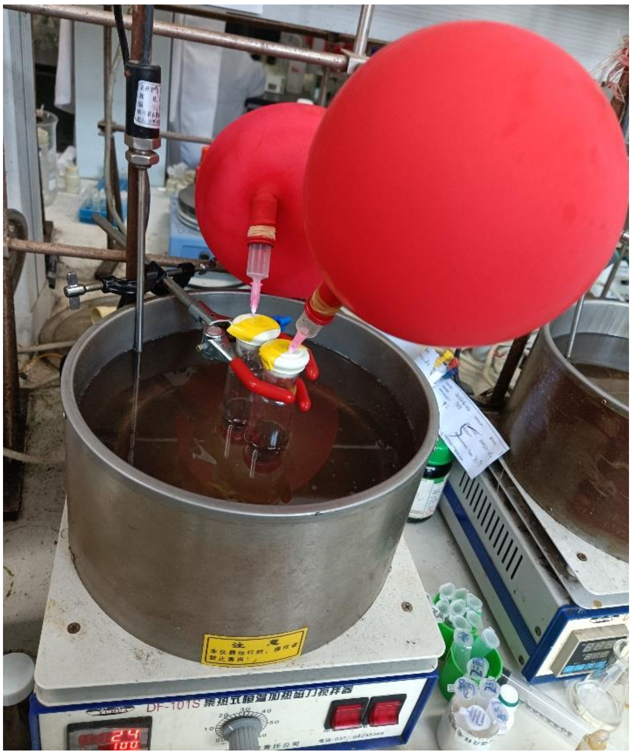

Figure S5: Seal the pinholes with insulative tape and vacuum grease.

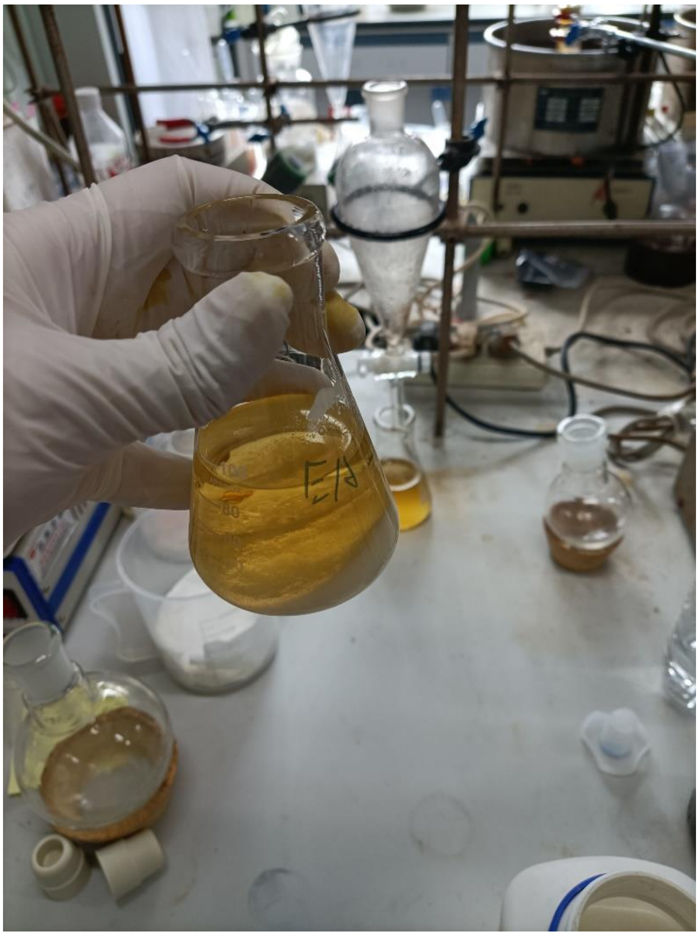

Figure S6: After the reaction is finished, extract with EA/water, take the EA layer and dry with anhydrous Na_2SO_4.

3.3 General procedures of mechanism investigation (a)

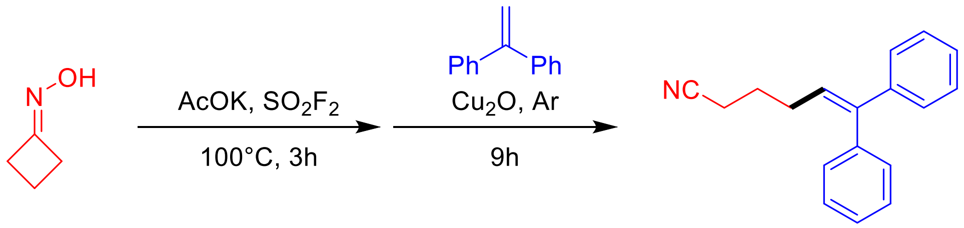

AcOK (10.0 mmol), cyclobutanone oxime (3.0 mmol), and extra dry 1,4-dioxane (10 mL) were added to a 50 mL oven-dried round-bottomed flask that was equipped with a stirring bar. The flask was fitted with a plastic stopper and SO_2F_2 gas was introduced into the stirring reaction mixture by bubbling from a SO_2F_2 balloon. The mixture was stirred strongly at 100 °C for 3 h, then Cu_2O (1.0 mmol), 1,1-diphenylethylene (1.0 mmol) were added to the reaction mass. Argon was introduced into the stirring reaction mixture by bubbling from an argon balloon. Keep stirred at 100 °C for another 9 h. After that, the yield of the product was detected by HPLC using pure **3aa** as the external standard (t_R = 5.017 min, λ_{max} = 250.0 nm, water/methanol 20:80 (v/v)), giving a yield of 45%.

3.4 General procedures of mechanism investigation (b)

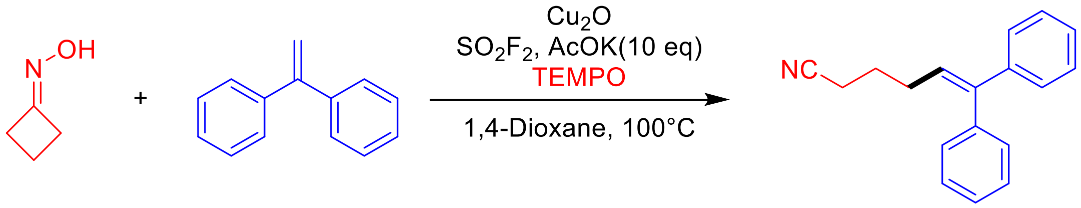

Cu_2O (1.0 mmol), AcOK (10.0 mmol), cyclobutanone oxime (3.0 mmol), 1,1-diphenylethylene (1.0 mmol), TMEPO (1.0 or 2.0 mmol) and extra dry 1,4-dioxane (10 mL) were added to a 50 mL oven-dried round-bottomed flask that was equipped with a stirring bar. The flask was fitted with a plastic stopper and SO_2F_2 gas was introduced into the stirring reaction mixture by bubbling from a SO_2F_2 balloon. The mixture was stirred strongly at 100 °C for 12 h, then cooled to room temperature upon completion. After that, the yield of the reaction was detected by HPLC using pure **3aa** as the external standard (t_R = 5.017 min, λ_{max} = 250.0 nm, water/methanol 20:80 (v/v)).

4. Product characterization

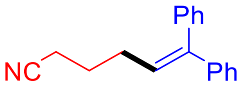

3aa

6,6-Diphenylhex-5-enenitrile **(3aa)**

General procedures for synthesis of the compounds 3, Method A: **3aa** (206 mg, 83%), yellow oil. The NMR data is identical to that reported in literature[3]. ^{1}H NMR (500 MHz, $CDCl_3$) δ 7.41 – 7.38 (m, 2H), 7.35 – 7.32 (m, 1H), 7.29 – 7.22 (m, 5H), 7.17 – 7.16 (m, 2H), 6.02 (t, J = 7.5 Hz, 1H), 2.33 – 2.25 (m, 4H), 1.84 – 1.78 (m,2H);

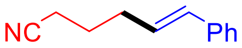

3ab

(E)-6-Phenylhex-5-enenitrile **(3ab)**

General procedures for synthesis of the compounds 3, Method B: **3ab** (117 mg, 68%), yellow oil. The NMR data is identical to that reported in literature[3]. ^{1}H NMR (500 MHz, $CDCl_3$) δ 7.36 – 7.30 (m, 5H), 6.47 (d, J = 16.0 Hz, 1H), 6.17 – 6.11 (m, 1H), 2.41 – 2.38 (m, 4H), 1.89 – 1.83 (m, 2H);

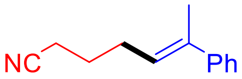

3ac

(E)-6-Phenylhept-5-enenitrile **(3ac)**

General procedures for synthesis of the compounds 3, Method A: **3ac** (121 mg, 66%), yellow oil. The NMR data is identical to that reported in literature[3]. ^{1}H NMR (500 MHz, $CDCl_3$) δ 7.38 – 7.37 (m, 2H), 7.34 – 7.31 (m, 2H), 7.25 – 7.22 (m, 1H), 5.71 – 5.68 (m, 1H), 2.42 – 2.37 (m, 4H), 2.07 (s, 3H), 1.87 – 1.81 (m, 2H);

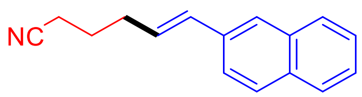

3ad

***(E)-6-(Naphthalen-2-yl)hex-5-enenitrile* (3ad)**

General procedures for synthesis of the compounds 3, Method A: **3ad** (125 mg, 56%), white solid. The NMR data is identical to that reported in literature[4]. ^{1}H NMR (500 MHz, $CDCl_3$) δ 7.81 – 7.78 (m, 3H), 7.70 (s, 1H), 7.57 (dd, J_1 = 8.5 Hz, J_2 = 1.5 Hz, 1H), 7.47 – 7.44 (m, 2H), 6.63 (d, J = 16.0 Hz, 1H), 6.30 – 6.24 (m, 1H), 2.47 – 2.41 (m, 4H), 1.92 – 1.86 (m, 2H);

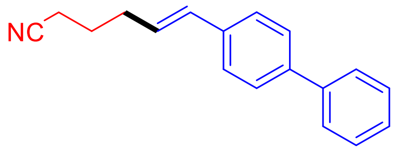

3ae

***(E)-6-([1,1'-Biphenyl]-4-yl)hex-5-enenitrile* (3ae)**

General procedures for synthesis of the compounds 3, Method A: **3ae** (153 mg, 62%), yellow solid. The NMR data is identical to that reported in literature[5]. ^{1}H NMR (500 MHz, $CDCl_3$) δ 7.60 (d, *J* = 7.5 Hz, 2H), 7.56 (d, *J* = 8.0 Hz, 2H), 7.46 – 7.43 (m, 4H), 7.35 (t, *J* = 7.5 Hz, 1H), 6.51 (d, *J* = 15.5 Hz, 1H), 6.22 – 6.16 (m, 1H), 2.44 – 2.40 (m, 4H), 1.90 – 1.85 (m, 2H);

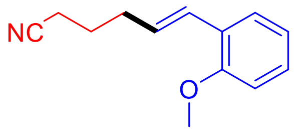

3af

***(E)-6-(2-Methoxyphenyl)hex-5-enenitrile* (3af)**

General procedures for synthesis of the compounds 3, Method B: **3af** (125 mg, 62%), yellow oil. The NMR data is identical to that reported in literature[5]. ^{1}H NMR (500 MHz, $CDCl_3$) δ 7.42 – 7.40 (m, 1H), 7.24 – 7.20 (m, 1H), 6.93 (t, J = 7.5 Hz, 1H), 6.87 (d, J = 8.0 Hz, 1H), 6.78 (d, J = 16.0 Hz, 1H), 6.17 – 6.11 (m, 1H), 3.85 (s, 3H), 2.42 – 2.38 (m, 4H), 1.89-1.83 (m, 2H).

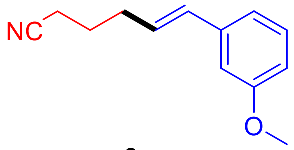

3ag

(E)-6-(3-Methoxyphenyl)hex-5-enenitrile **(3ag)**

General procedures for synthesis of the compounds 3, Method B: **3ag** (106 mg, 52%), yellow oil. The NMR data is identical to that reported in literature[5]. ^{1}H NMR (500 MHz, $CDCl_3$) δ 7.23 (t, J = 8.0 Hz, 1H), 6.95 (d, J = 7.5 Hz,1H), 6.89 (s , 1H), 6.80 – 6.78 (m, 1H), 6.44 (d, J = 16.0 Hz, 1H), 6.16 – 6.10 (m, 1H), 3.82 (s, 3H), 2.41 – 2.37 (m, 4H), 1.88 – 1.83 (m, 2H).

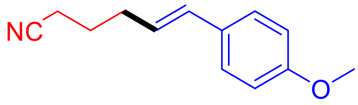

3ah

(E)-6-(4-Methoxyphenyl)hex-5-enenitrile **(3ah)**

General procedures for synthesis of the compounds 3, Method B: **3ah** (128 mg, 63%), yellow oil. The NMR data is identical to that reported in literature[5]. ^{1}H NMR (500 MHz, $CDCl_3$) δ 7.29 – 7.28 (m, 2H), 6.87 – 6.84 (m, 2H), 6.40 (d, J = 16.0 Hz, 1H), 6.01 – 5.95 (m, 1H), 3.80 (s, 3H), 2.40 – 2.34 (m, 4H), 1.86 – 1.81 (m, 2H).

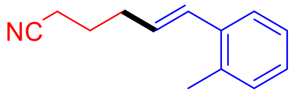

3ai

(E)-6-(2-Methylphenyl)hex-5-enenitrile **(3ai)**

General procedures for synthesis of the compounds 3, Method B: **3ai** (118 mg, 64%), yellow oil. The NMR data is identical to that reported in literature[5]. ^{1}H NMR (500 MHz, $CDCl_3$) δ 7.41 – 7.40 (m, 1H), 7.16 – 7.04 (m, 3H), 6.68 (d, J = 16.0 Hz, 1H), 6.03 – 5.97 (m, 1H), 2.43 – 2.40 (m, 4H), 2.35 (s, 3H), 1.90 – 1.84 (m, 2H);

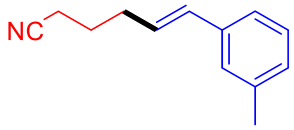

3aj

***(E)*-6-(3-Methylphenyl)hex-5-enenitrile** (3aj)

General procedures for synthesis of the compounds 3, Method B: **3aj** (102 mg, 55%), yellow oil. The NMR data is identical to that reported in literature.[5] **^{1}H NMR (500 MHz, $CDCl_3$)** δ 7.10 (t, J = 7.5 Hz, 1H), 7.07 – 7.04(m, 2H), 6.96 – 6.93 (m, 1H), 6.34 (d, J = 15.5 Hz, 1H), 6.05 – 5.99 (m, 1H), 2.30 – 2.28 (m, 4H), 2.25 (s, 3H), 1.78 – 1.72 (m, 2H);

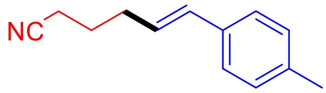

3ak

***(E)*-6-(4-Methylphenyl)hex-5-enenitrile** (3ak)

General procedures for synthesis of the compounds 3, Method B: **3ak** (67 mg, 36%), yellow oil. The NMR data is identical to that reported in literature[5]. ^{1}H NMR (500 MHz, $CDCl_3$) δ 7.24 (d, J = 8.0 Hz, 2H), 7.10 (d, J = 10.0 Hz, 2H), 6.43 (d, J = 16.0 Hz, 1H), 6.10 – 6.04 (dt, J_1 = 15.5 Hz, J_2 = 7.5 Hz, 1H), 2.40 – 2.37 (m, 4H), 2.33 (s, 3H), 1.87 – 1.82 (m, 2H);

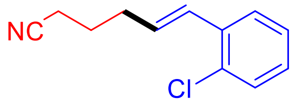

3al

***(E)*-6-(2-Chlorophenyl)hex-5-enenitrile** (3al)

General procedures for synthesis of the compounds 3, Method B: **3al** (41 mg, 20%), yellow oil. The NMR data is identical to that reported in literature[5]. ^{1}H NMR (500 MHz, $CDCl_3$) δ 7.49 (dd, J_1 = 8.0 Hz, J_2 = 2.0 Hz, 1H), 7.36 – 7.35 (m, 1H), 7.21 – 7.15 (m, 2H), 6.83 (d, J = 16.0 Hz, 1H), 6.12 (dt, J_1 = 16.0 Hz, J_2 = 7.0 Hz, 1H), 2.44 – 2.40 (m, 4H), 1.90 – 1.87 (m, 2H);

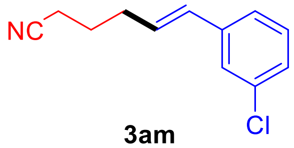

3am

(E)-6-(3-Chlorophenyl)hex-5-enenitrile **(3am)**

General procedures for synthesis of the compounds 3, Method B: **3am** (66 mg, 32%), yellow oil. The NMR data is identical to that reported in literature[5]. ^{1}H NMR (500 MHz, $CDCl_3$) δ 7.33 (s, 1H), 7.25 – 7.17 (m, 3H), 6.41 (d, J = 16.0 Hz, 1H), 6.15 (dt, J_1 = 15.5 Hz, J_2 = 7.0 Hz, 1H), 2.42 – 2.37 (m, 4H), 1.88 – 1.82 (m, 2H);

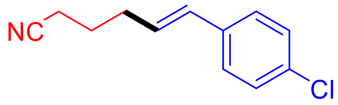

3an

(E)-6-(4-Chlorophenyl)hex-5-enenitrile **(3an)**

General procedures for synthesis of the compounds 3, Method B: **3an** (98 mg, 48%), yellow oil. The NMR data is identical to that reported in literature[5]. ^{1}H NMR (500 MHz, $CDCl_3$) δ 7.31 – 7.24 (m, 4H), 6.42 (d, *J* = 16.0 Hz, 1H), 6.14 – 6.10 (m, 1H), 2.41 – 2.37 (m, 4H), 1.88 – 1.84 (m, 2H);

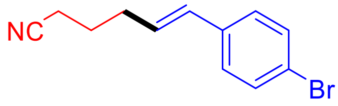

3ao

(E)-6-(4-Bromophenyl)hex-5-enenitrile **(3ao)**

General procedures for synthesis of the compounds 3, Method B: **3ao** (105 mg, 42%), yellow oil. The NMR data is identical to that reported in literature[5]. ^{1}H NMR (500 MHz, $CDCl_3$) δ 7.46 – 7.39 (m, 2H), 7.23 – 7.18 (m, 2H), 6.40 (d, *J* = 16.0 Hz, 1H), 6.16 – 6.09 (m, 1H), 2.41 – 2.36 (m, 4H), 1.87 – 1.84 (m, 2H);

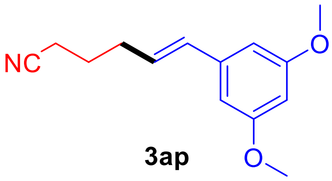

3ap

***(E)-6-(3,5-Dimethoxyphenyl)hex-5-enenitrile* (3ap)**

General procedures for synthesis of the compounds 3, Method B: **3ap** (144 mg, 62%), yellow oil. The NMR data is identical to that reported in literature[6]. ^{1}H NMR (500 MHz, $CDCl_3$) 6.98 (s, 2H), 6.89 (s, 1H), 6.41 (d, J = 16.0 Hz, 1H), 6.14 – 6.08 (m, 1H), 2.41 – 2.36 (m, 4H), 2.31 (s, 6H), 1.88 – 1.82 (m, 2H);

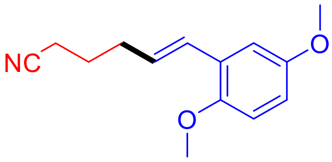

3aq

***(E)-6-(2,5-Dimethoxyphenyl)hex-5-enenitrile* (3aq)**

General procedures for synthesis of the compounds 3, Method B: **3aq** (119 mg, 51%), yellow oil. The NMR data is identical to that reported in literature[6]. ^{1}H NMR (500 MHz, $CDCl_3$) 6.96 (d, J = 3.0 Hz, 1H), 6.81 – 6.73 (m, 3H), 6.16 – 6.10 (m, 1H), 3.81 (s, 3H), 3.79 (s, 3H),2.42 – 2.38 (m, 4H), 1.89 – 1.83 (m, 2H);

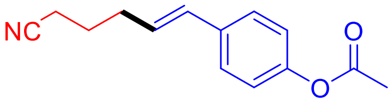

3ar

***(E)-4-(5-Cyanopent-1-en-1-yl)phenyl acetate* (3ar)**

General procedures for synthesis of the compounds 3, Method B: **3aq** (100 mg, 44%), yellow oil. The NMR data is identical to that reported in literature[4]. ^{1}H NMR (500 MHz, $CDCl_3$) 7.35 (d, J = 8.5 Hz, 2H), 7.03 (d, J = 8.5 Hz, 2H), 6.45 (d, J = 16.0 Hz, 1H), 6.11 – 6.05 (m, 1H), 2.40 – 2.38 (m, 4H), 2.30 (s, 3H), 1.87 – 1.82 (m, 2H);

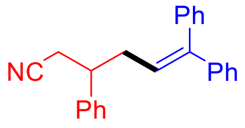

3ba

***3,6,6-Triphenylhex-5-enenitrile* (3ba)**

General procedures for synthesis of the compounds 3, Method A: **3ba** (243 mg, 75%), yellow oil. The NMR data is identical to that reported in literature[3]. ^{1}H NMR (500 MHz, $CDCl_3$) δ 7.43 – 7.37 (m, 5H), 7.33 – 7.25 (m, 4H), 7.20 – 7.15 (m, 4H), 7.11 – 7.10 (m, 2H), 5.96 (t, *J* = 7.5 Hz, 1H), 3.16 – 3.13 (m, 1H), 2.68 – 2.56 (m, 4H);

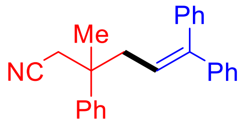

3ca

***3-Methyl-3,6,6-triphenylhex-5-enenitrile* (3ca)**

General procedures for synthesis of the compounds 3, Method A: **3ca** (236 mg, 70%), yellow oil. The NMR data is identical to that reported in literature[3]. ^{1}H NMR (500 MHz, $CDCl_3$) δ 7.41 – 7.35 (m, 5H), 7.32 – 7.29 (m, 3H), 7.22 – 7.21 (m, 3H), 7.11 – 7.06 (m, 4H), 5.78 (t, J = 7.0 Hz, 1H), 2.70 – 2.67 (m, 2H), 2.61 – 2.57 (m, 2H), 1.55 (s, 3H);

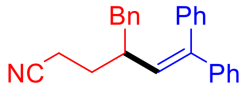

3da

***4-Benzyl-6,6-diphenylhex-5-enenitrile* (3da)**

General procedures for synthesis of the compounds 3, Method A: **3da** (273 mg, 81%), yellow oil. The NMR data is identical to that reported in literature[3]. ^{1}H NMR (500 MHz, $CDCl_3$) δ7.35 – 7.28 (m, 5H), 7.26 – 7.15 (m, 4H), 7.05 – 7.03 (m, 2H), 6.81 – 6.79 (m, 2H), 5.83 (d, J = 10.0 Hz, 1H), 2.76 – 2.66 (m, 2H), 2.55 – 2.51 (m,

1H), 2.38 – 2.32 (m, 1H), 2.19 – 2.12 (m, 1H), 1.85 – 1.81 (m, 1H), 1.71 – 1.65 (m, 1H).

5. References

1. Cho H; Iwama Y; Sugimoto K; Mori S; Tokuyama H. *J. Org. Chem.* **2010**; *75*: 627.
2. Xu H-J; Zhu F-F; Shen Y-Y; Wan X; Feng Y-S. *Tetrahedron.* **2012**; *68*: 4145.
3. Zhao B; Shi Z. *Angew. Chem. Int. Edit.* **2017**; *56*: 12727.
4. Lu X Y; Xia Z J; Gao A; Liu Q L; Jiang R C; Liu C C. *J. Org. Chem.* **2021**; *86*: 8829.
5. Gao J; Ye Z P; Liu Y F; He X C; Guan J P; Liu F; Chen K; Xiang H Y; Chen X Q; Yang H. *Org Lett.* **2022**; *24*: 4640.
6. Gigant N; Backvall J E. *Org Lett.* **2014**; *16*: 4432.

6. NMR spectra

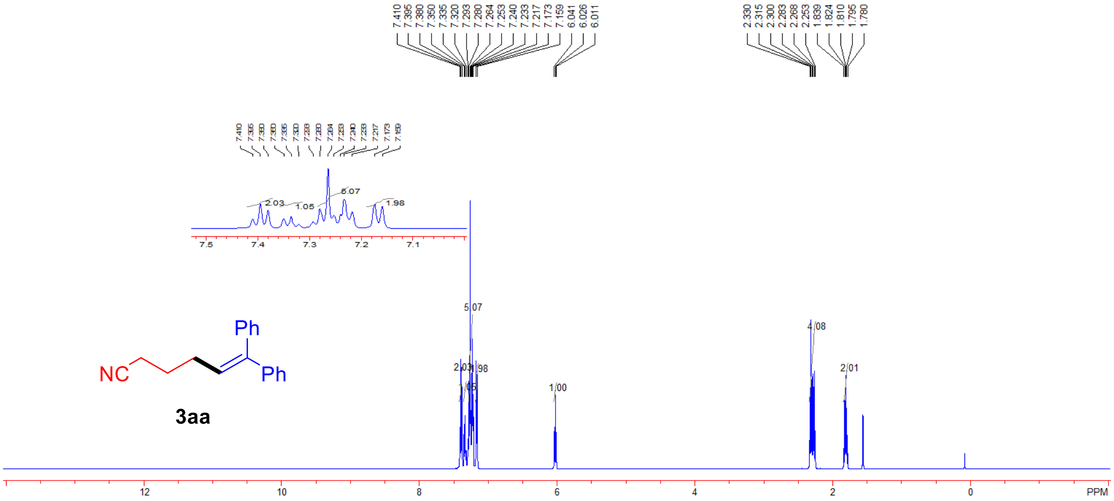

3aa

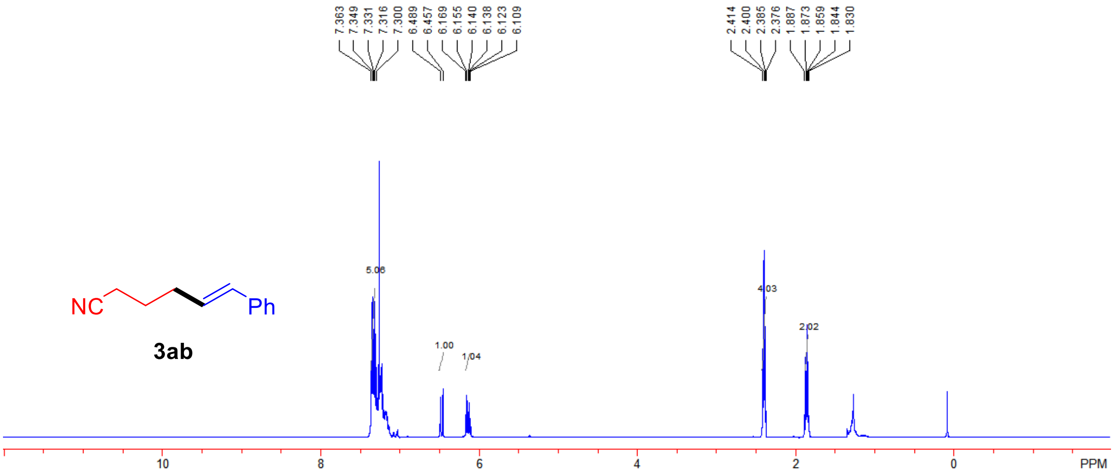

3ab

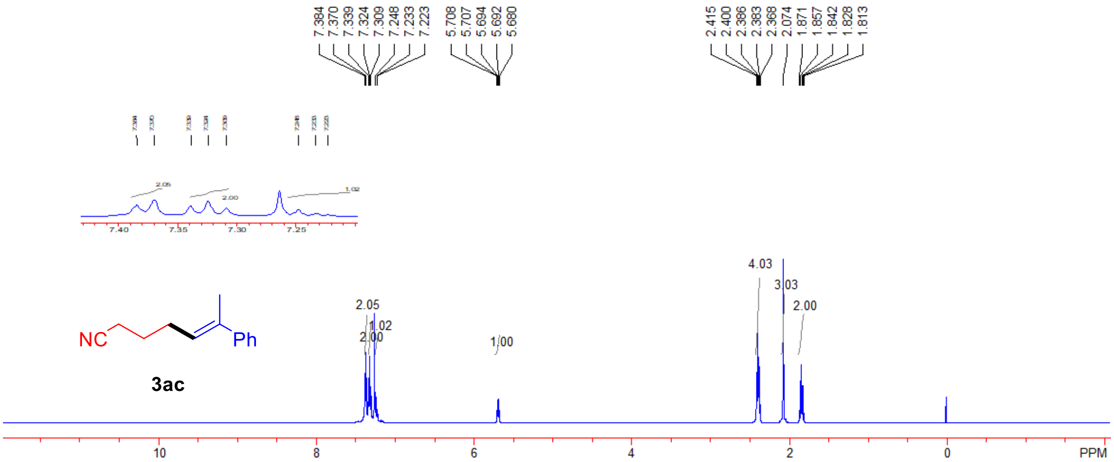

3ac

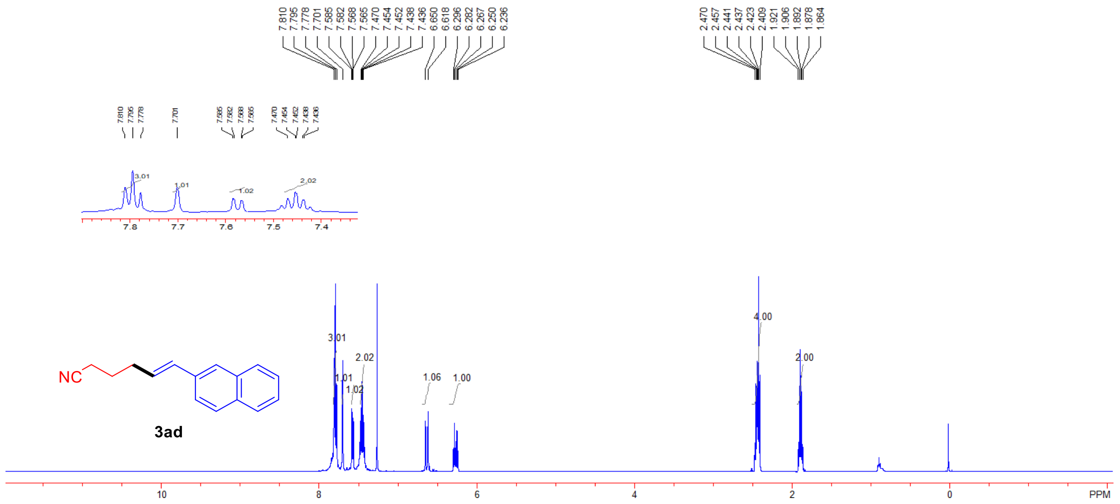
NC
3ad
3.01
1.01
1.02
2.02
1.06
1.00
4.00
2.00
PPM

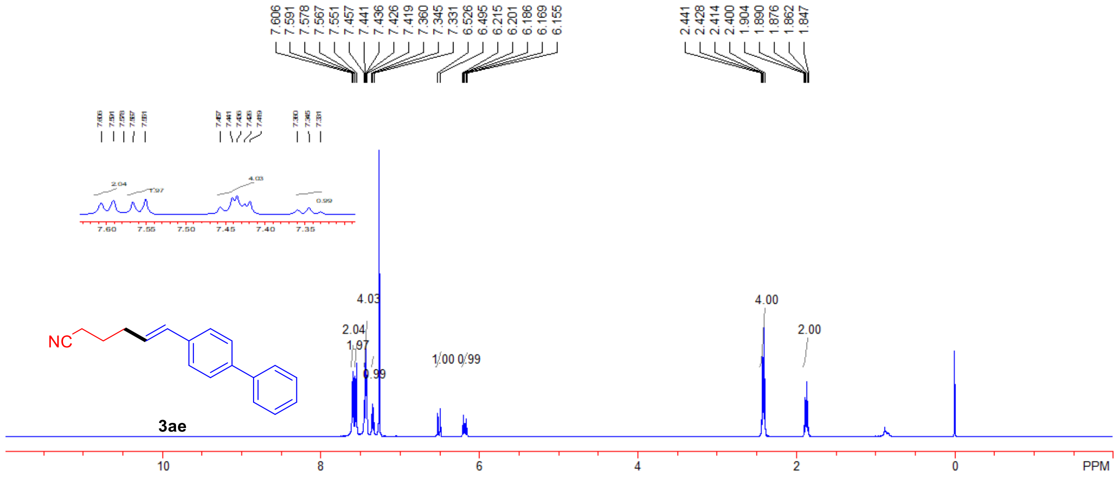
NC
3ae
2.04
1.97
4.03
0.99
1.00
0.99
4.00
2.00
PPM

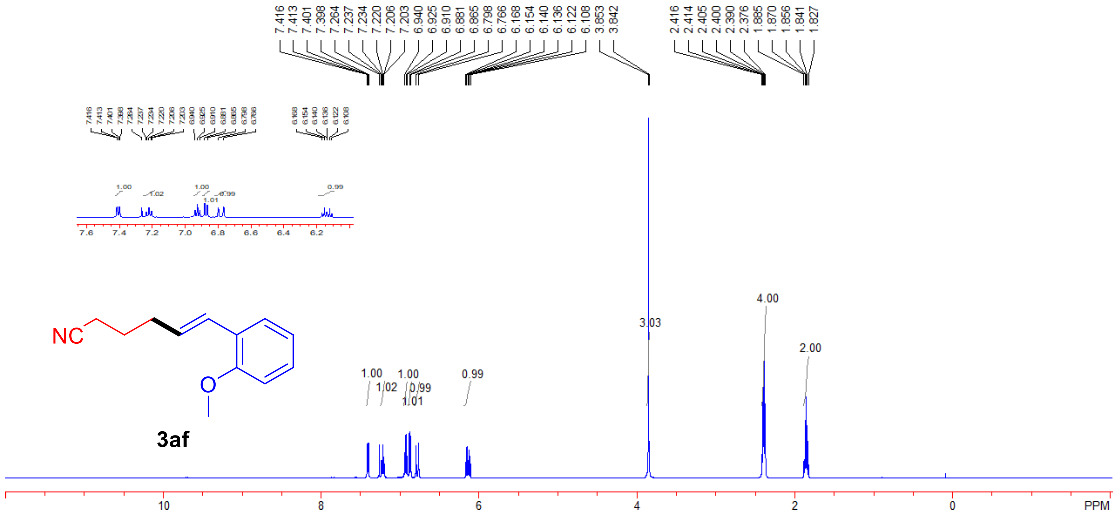
NC
O
3af
1.00
1.02
1.00
0.99
1.01
0.99
3.03
4.00
2.00
PPM

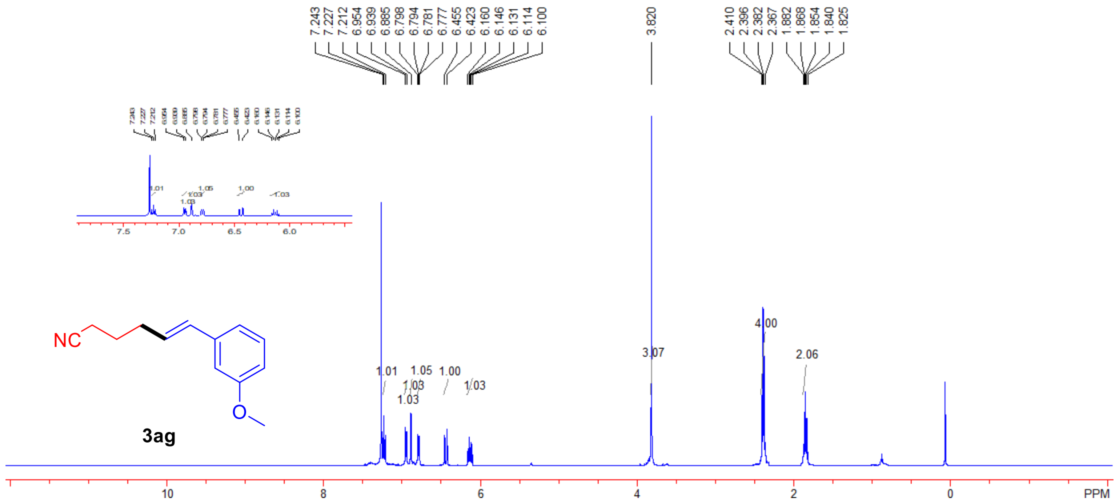

3ag

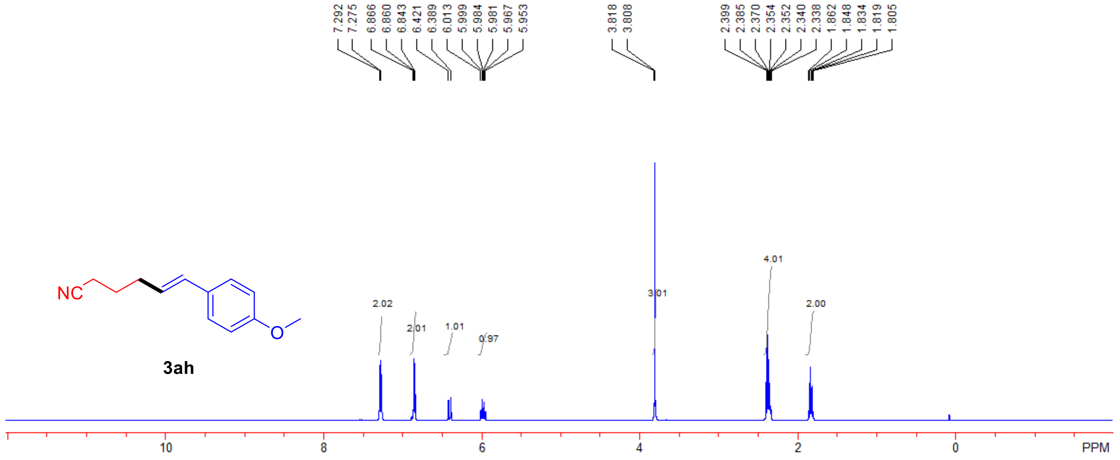

3ah

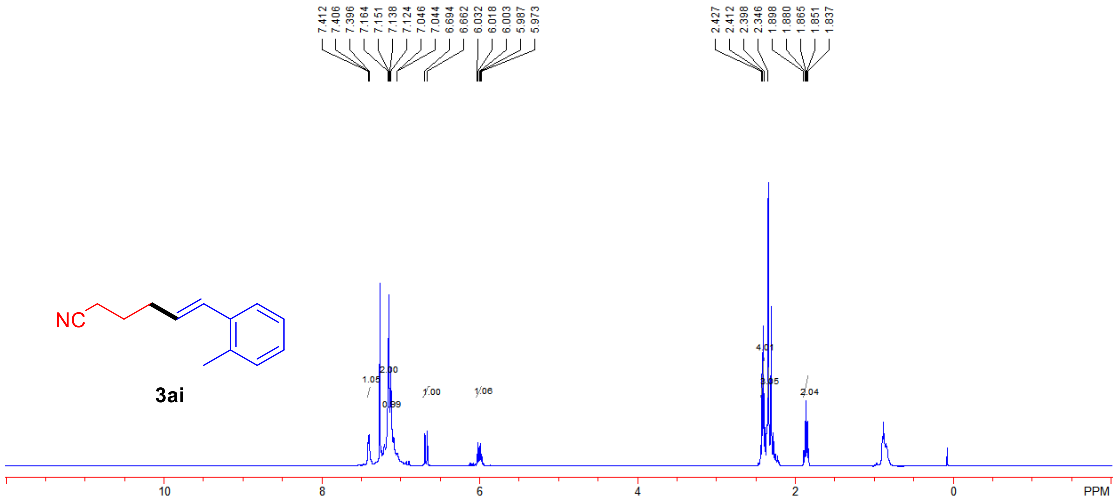

3ai

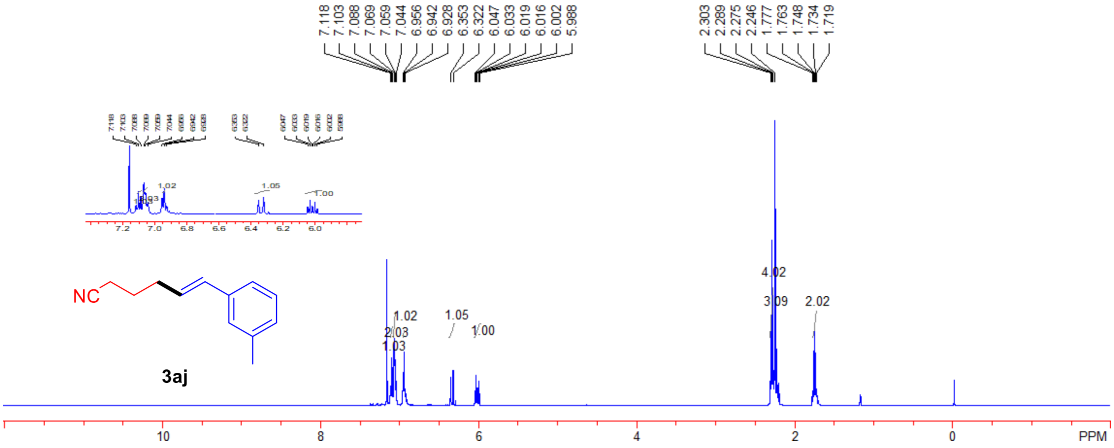

NC
3aj
PPM

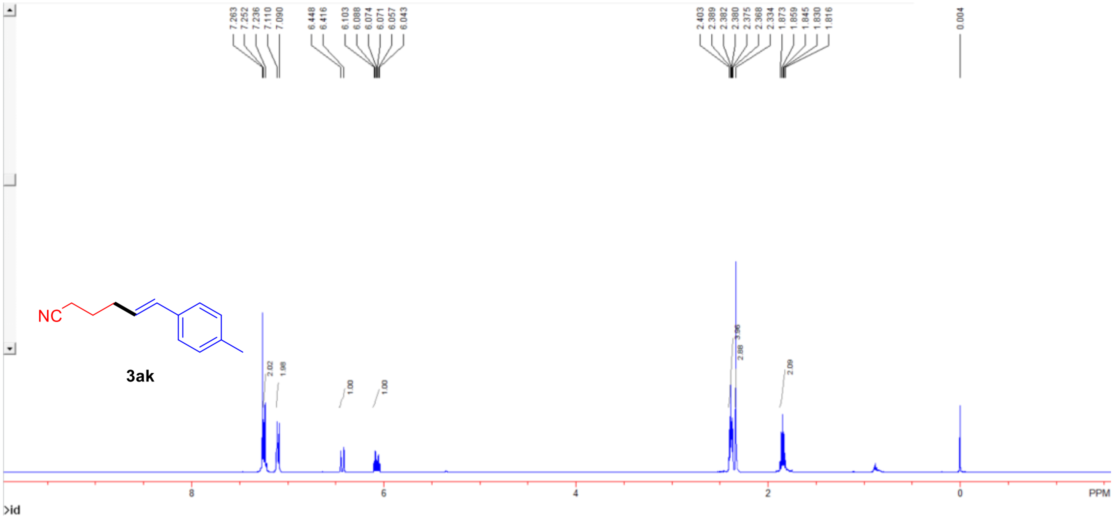

NC
3ak
PPM

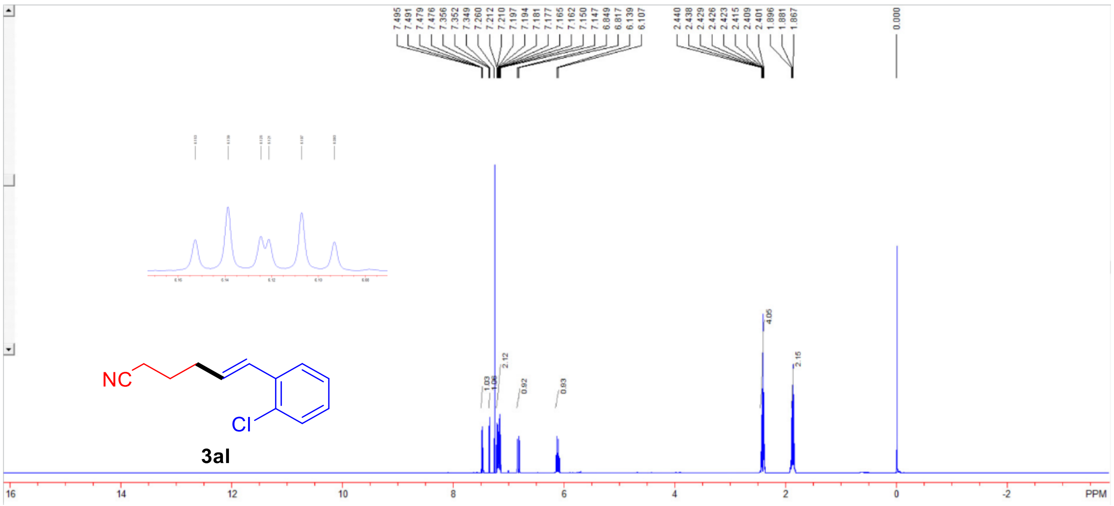

NC
Cl
3al
PPM

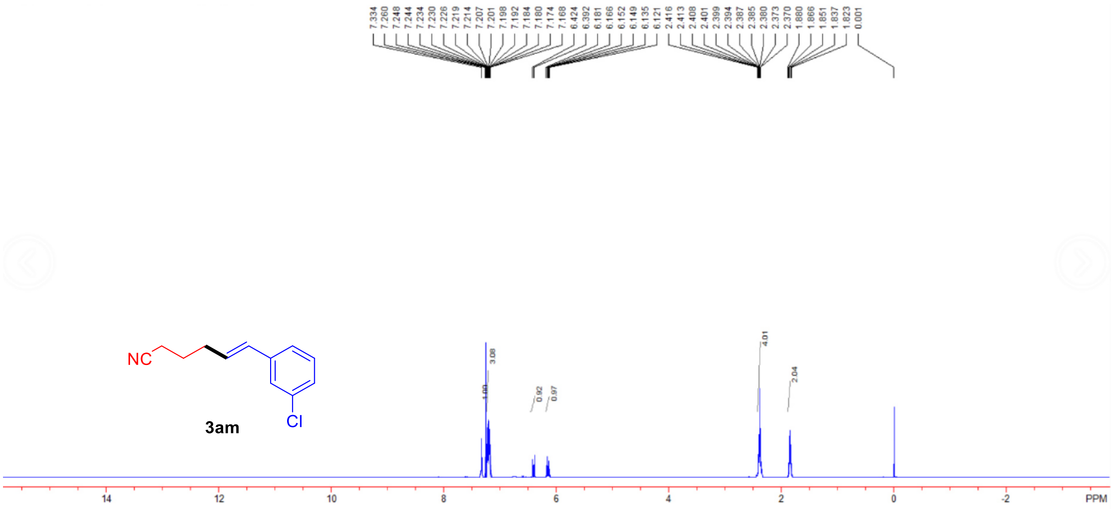

3am

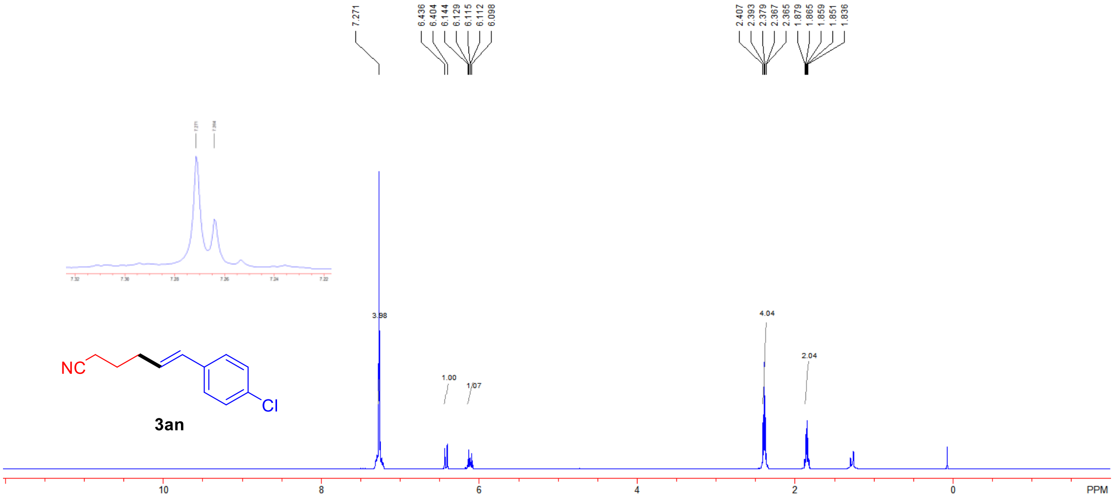

3an

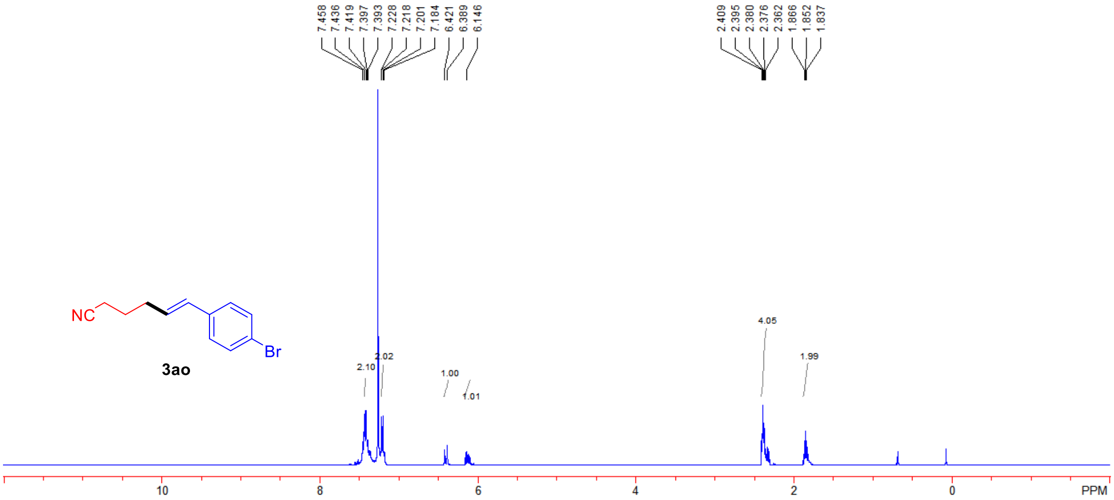

3ao

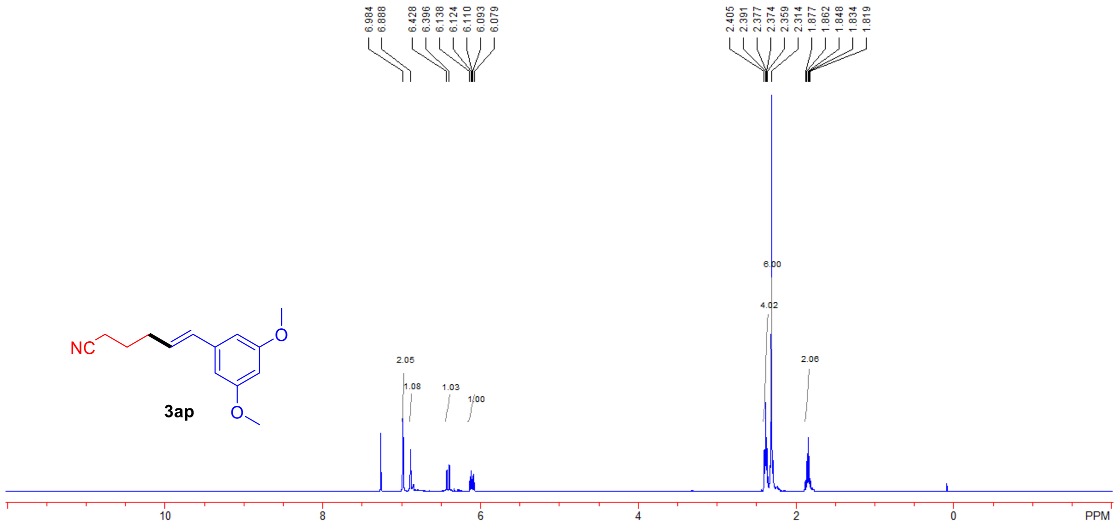

6.984
6.888
6.428
6.396
6.138
6.124
6.110
6.093
6.079
2.405
2.391
2.377
2.374
2.359
2.314
1.877
1.862
1.848
1.834
1.819
6.00
4.02
2.05
1.08
1.03
1.00
2.06
NC
O
O
10
8
6
4
2
0
PPM

3ap

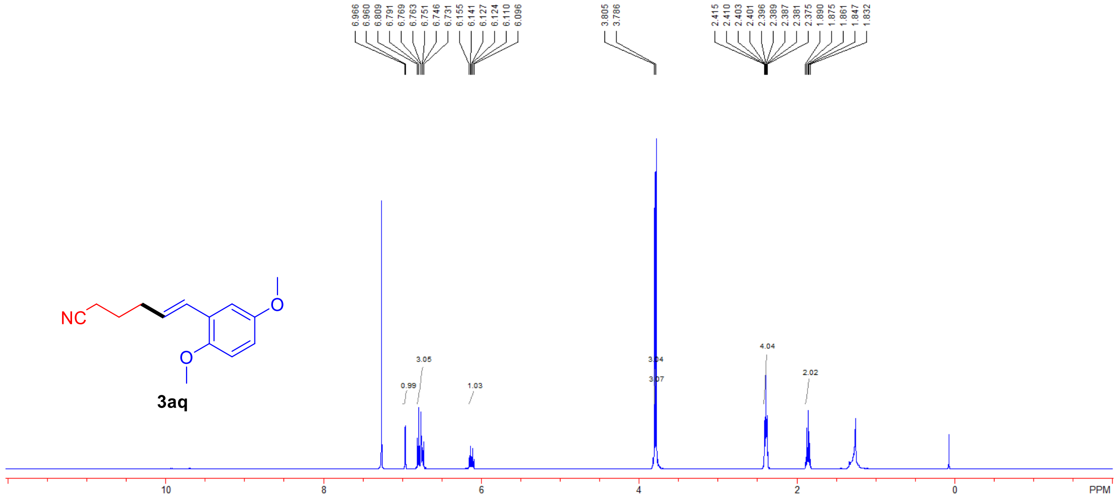

6.966
6.960
6.809
6.791
6.769
6.763
6.751
6.746
6.731
6.155
6.141
6.127
6.124
6.110
6.096
3.805
3.786
2.415
2.410
2.403
2.401
2.396
2.389
2.387
2.381
2.375
1.890
1.875
1.861
1.847
1.832
3.05
0.99
1.03
3.04
3.07
4.04
2.02
NC
O
O
10
8
6
4
2
0
PPM

3aq

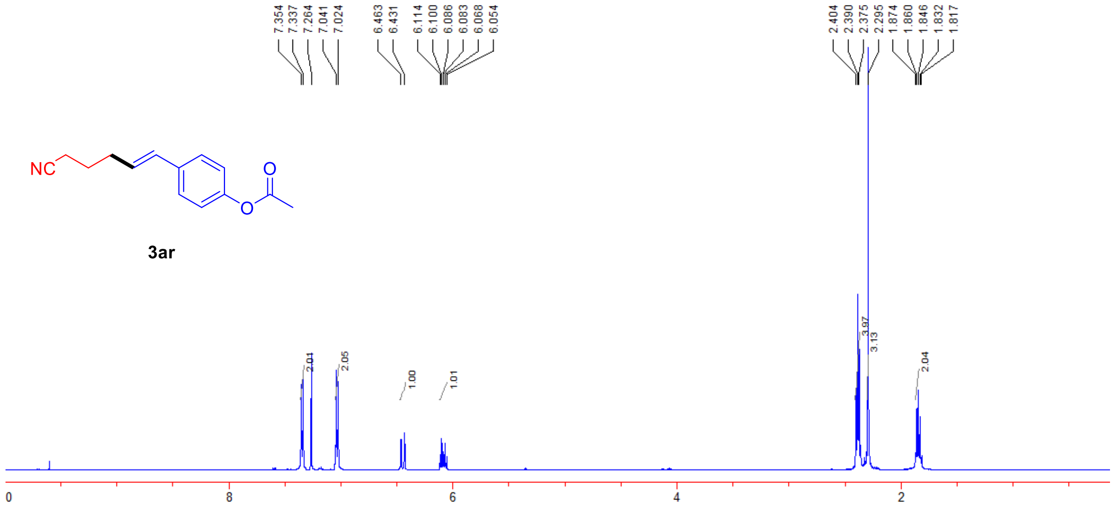

7.354
7.337
7.264
7.041
7.024
6.463
6.431
6.114
6.100
6.086
6.083
6.068
6.054
2.404
2.390
2.375
2.295
1.874
1.860
1.846
1.832
1.817
2.01
2.05
1.00
1.01
3.97
3.13
2.04
NC
O
O
0
8
6
4
2

3ar

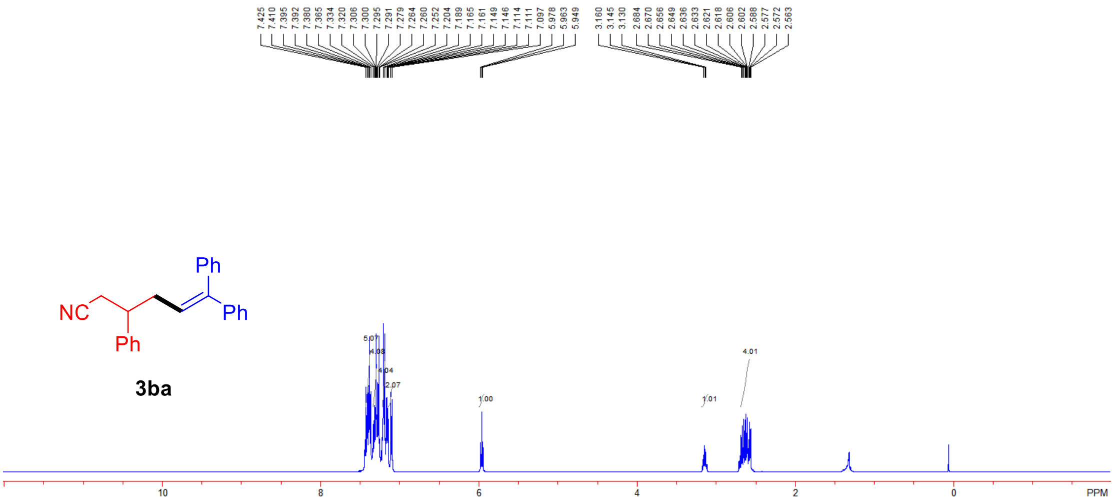

3ba

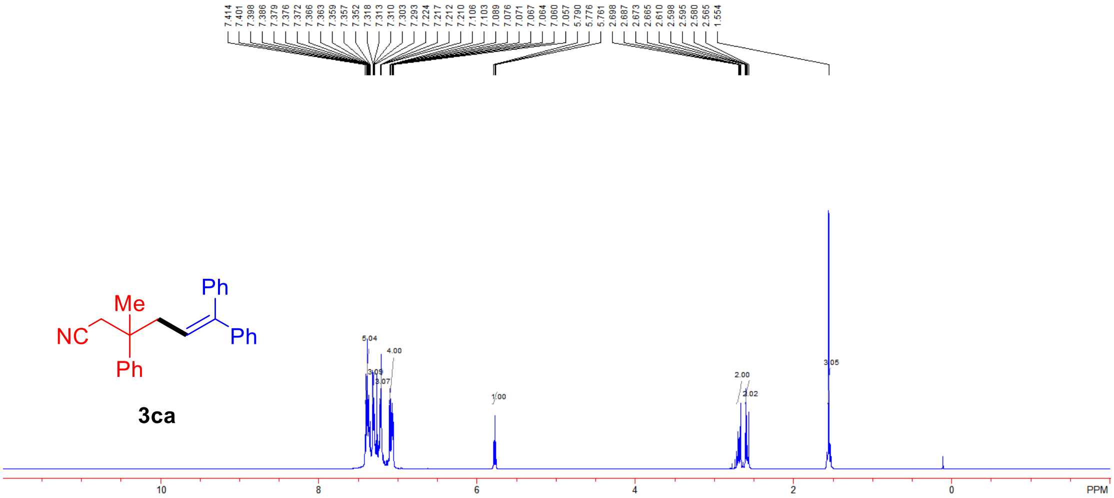

3ca

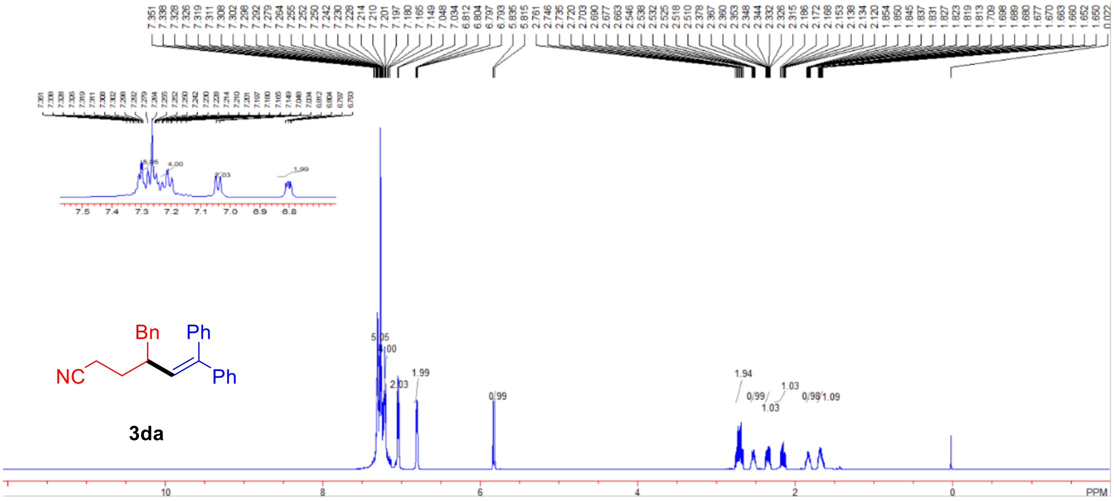

3da
